# Exposure to Mild Steel Welding and Changes in Serum Proteins With Putative Neurological Function—A Longitudinal Study

**DOI:** 10.3389/fpubh.2020.00422

**Published:** 2020-08-28

**Authors:** Anda R. Gliga, Tahir Taj, Karin Wahlberg, Thomas Lundh, Eva Assarsson, Maria Hedmer, Maria Albin, Karin Broberg

**Affiliations:** ^1^Institute of Environmental Medicine, Karolinska Institutet, Stockholm, Sweden; ^2^Occupational and Environmental Medicine, Department of Laboratory Medicine, Lund University, Lund, Sweden

**Keywords:** neurotoxicity, particle, manganese, Parkinson, NMNAT1

## Abstract

Welders are exposed to high levels of metal particles, consisting mainly of iron and manganese (Mn) oxide. Metal particles, especially those containing Mn can be neurotoxic. In this exploratory study, we evaluated associations between welding and expression of 87 putative neurology-related proteins in serum in a longitudinal approach. The study cohort from southern Sweden included welders working with mild steel (*n* = 56) and controls (*n* = 67), all male and non-smoking, which were sampled at two timepoints (T1, T2) 6-year apart. Observed associations in the longitudinal analysis (linear mixed models) were further evaluated (linear regression models) in another cross-sectional sample which included welders (*n* = 102) and controls (*n* = 89) who were sampled only once (T1 or T2). The median respirable dust levels for welders after adjusting for respiratory protection was at T1 0.6 (5–95 percentile: 0.2–4.2) and at T2 0.5 (0.1–1.8) mg/m^3^. The adjusted median respirable Mn concentration was at T2 0.049 mg/m^3^ (0.003–0.314) with a Spearman correlation between adjusted respirable dust and respirable Mn of *r*_S_ = 0.88. We identified five neurology-related proteins that were differentially expressed in welders vs. controls in the longitudinal sample, of which one (nicotinamide/nicotinic acid mononucleotide adenylyltransferase 1; NMNAT1) was also differentially expressed in the cross-sectional sample. NMNAT1, an axon-protective protein linked to Alzheimers disease, was upregulated in welders compared with controls but no associations were discerned with degree of exposure (welders only: years welding, respirable dust, cumulative exposure). However, we identified five additional proteins that were associated with years welding (GCSF, EFNA4, CTSS, CLM6, VWC2; welders only) both in the longitudinal and in the cross-sectional samples. We also observed several neurology-related proteins that were associated with age and BMI. Our study indicates that low-to-moderate exposure to welding fumes is associated with changes in circulating levels of neurology-related proteins.

## Introduction

It is well-established that high exposure to manganese (Mn) is neurotoxic. Mn can accumulate in the brain and cause the condition manganism, a neurological syndrome with symptoms similar to Parkinson's disease (referred to as Parkinsonism) including tremor, body rigidness, reduced smell, and impaired motor function and balance (1). Exposure to Mn has also been associated with Alzheimer's disease in a study combining human, *in vitro* and animal experiments (2). Welders have for a long time been considered a risk group of high Mn exposure due to the release of respirable Mn particles during welding (3, 4). Several studies have indicated that exposure to Mn in occupational settings is associated with neurological and neuropsychological health effects, higher prevalence of Parkinsonism, and increased Mn in brain as observed by magnetic resonance imaging scans (5–7), whereas other studies that have found no association (8, 9).

In Sweden, around 13,000 workers are registered as full-time welders of steel (10). Measurements during 2003 to 2005 at 10 different Swedish workplaces revealed that many welders were exposed to Mn levels above the occupational exposure limit (OEL) at that time (0.1 mg/m^3^) and that the average exposure of all welders (0.08 mg/m^3^, *n* = 108) was near the OEL (11). It was also observed that welders were still performing a major part of the work without appropriate protection (12). The current OEL for Mn in Sweden is 0.05 mg/m^3^ (8-h total weight average - TWA) as respirable fraction (13). This is in line with the OEL in the European Union (14) while other countries, such as Germany and Finland, have a lower OEL of Mn in the respirable fraction (0.02 mg/m^3^) (13).

In addition to Mn ions, exposure to metal nanoparticles in the welding fumes may also cause neurotoxic effects. *In vitro* studies indicate that neurotoxicity (e.g., cell death and oxidative stress) in neuronal cell models can be induced by both Mn oxide nanoparticles (15) and iron oxide nanoparticles (16, 17). Evidence suggests that following inhalation, metal (nano)particles are able to directly translocate to the brain via the olfactory bulb and sensory nerve endings in the respiratory tract (18–20).

It is important to clarify if current exposure levels to welding fumes cause neurotoxicity as well as to identify biomarkers of neurological changes. In this study, we investigated if welders show alterations in serum levels of a panel of proteins putatively related to neurobiological processes and neurological diseases.

## Methods and Materials

### Study Design

This study is based on a cohort of welders established during 2010-2011 (timepoint 1) in the south of Sweden (21). The inclusion criteria were that the participants should be males and non-smokers for the last 6 months. At baseline, we recruited 101 welders working in 10 small- and medium-size welding companies and 127 age-matched controls working in medium-size companies (mainly food business) and municipalities (working as janitors or gardeners), all without occupational exposure to welding fumes or particles from other sources. The follow-up after 6 years was performed during 2016–2017 (timepoint 2) and included welders from 9 of the initial 10 companies (one of the companies had closed). There was a drop-out of 23% (*n* = 23) among the welders and 24% (*n* = 31) among the controls at timepoint 2 and the reasons were: sick on the medical examination day, declined participation without giving any particular reason, or had died since timepoint 1. At timepoint 2, we also recruited new participants (67 welders and 38 controls) with the same inclusion criteria. Overall, the timepoint 2 survey included examination of 145 welders (78 welders re-examined) and 134 controls (96 controls re-examined). Among welders participating at both timepoints, í out of 78 had retired or quit working due to illness since timepoint 1, and another 12 were employed at the same company as before but were no longer actively involved in welding. Those seven that had quit working were invited to visit either their old company or the clinic for the follow-up examination.

**Figure 1:**
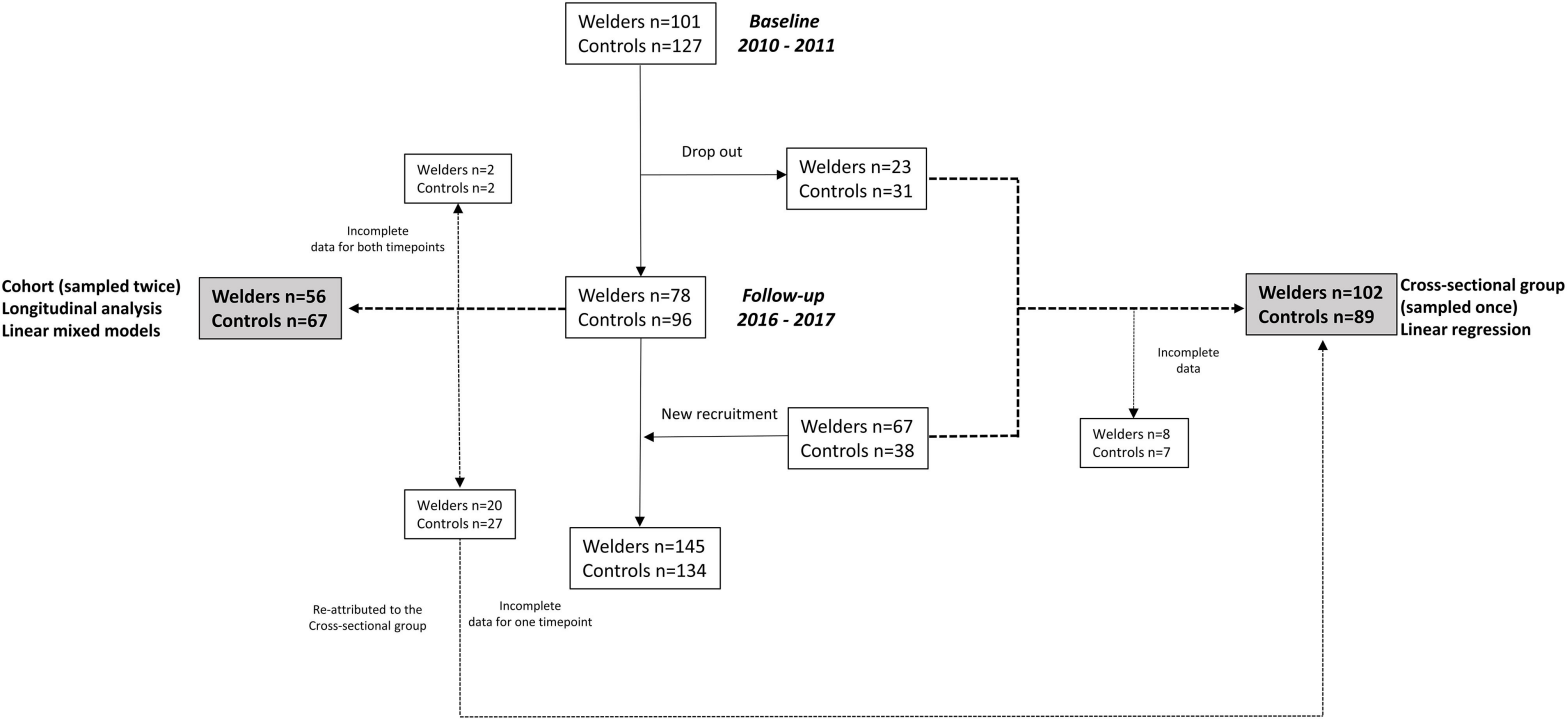
Flow-chart of the study design. Incomplete data refers to low quality of the protein data.

In this study, the participants were divided into two groups: one group with repeated measurements 2010/2011 and 2016/2017, and one group with measurements performed at either 2010/2011 or 2016/2017, i.e., cross-sectional group (90 welders and 69 controls). The latter group was used to see if we could replicate the associations observed in the group with longitudinal data. In the cross-sectional group, 8 welders and 7 controls were excluded from the analysis due to missing/poor quality data for the protein measurements. In addition, 27 controls and 20 welders were re-attributed to the cross-sectional group due to missing/poor quality data for the protein measurements for one of the timepoints. Further, 2 welders as well as 2 controls were excluded due to missing/poor quality data for the protein measurements at both timepoints. This amounts to a total of 123 individuals in the cohort group (56 welders and 67 controls) and 191 individuals in the cross-sectional group (102 welders and 89 controls). A flow diagram of the study design is shown in Figure 1. A similar study design on the same cohort of Swedish welders was used to evaluate the associations between cancer-related proteins in serum (22).

The participants filled out a questionnaire regarding country of birth, education, medical history, personal/family history of cancer, diet, physical activity, current as well as previous smoking history, use of snus (Swedish moist tobacco), alcohol consumption, current residence, and exposure to particles/smoke (e.g., welding fumes, dust, engine exhaust, diesel engine) during leisure activities. The inclusion criteria were that the participants should be non-smokers for the last 6 months. However, based on the questionnaire data, we identified at follow-up a few individuals that actually were current smokers.

Blood samples were collected in the same way at both timepoints in BD vacutainers for serum, allowed to clot at room temperature for 10 min and then centrifuged at 2,400 rcf for 10 min. Upon separation, serum samples were aliquoted and kept on dry ice for transportation to the laboratory in the Division of Occupational and Environmental Medicine at Lund University, and then stored at −80°C until analysis. Samples were shipped on dry ice to Uppsala for protein analysis.

### Exposure Assessment

Both for welders and controls, we used a structured questionnaire inquiring about the present and past workplaces, type and duration of work, and explicitly whether they were exposed to welding or diesel fumes at past or present workplaces. For welders, we also asked questions about the type of welding, total hours of welding during the usual working week, place of welding, area level or point source exhaust use, as well as use of personal respiratory, noise, and eye protection devices while welding.

### Personal Respirable Dust Measurement

Personal exposure measurement of respirable dust was performed for the active welders and stationary area-monitoring of respirable dust was conducted for the controls. For personal sampling, a cyclone (BGI4L, BGI, Mesa Labs, USA; cut-off = 4 μm) was used for collecting respirable dust. The cyclone was fitted with a filter cassette, containing 37-mm mixed cellulose ester filters with an 0.8-μm pore size (pre-weighed) and was placed within the breathing zone of each welder. The airflow through the sampler was set to 2.2 L/min and regularly checked before, during, and after sampling with a flow meter (TSI Model 4100 Series, TSI Incorporated, USA). Personal sampling was performed during one work day and was coordinated with each company's shift working hours: the average sampling duration was approximately 7 h for both timepoints. It was not feasible to perform measurements over multiple days due to the large number of participating welders. Measured dust concentrations were corrected if respiratory protection was used: the measured concentration (outside respiratory protection) was divided by 3 as a correction factor to reflect the actual exposure level (11, 21). At timepoint 2, one welder used a half-mask, for which a correction factor of 2 was used instead, and another four welders used newer versions of powered air-purifying respirators with double visors, for which a factor of 50 was used (personal communication with Karlsson J-E, occupational hygienist, Clinic of Occupational and Environmental Medicine, Lund University Hospital, Sweden). The filter samples were analyzed gravimetrically according to a validated method for determination of respirable dust (23). The limit of detection was set to 0.05 mg/sample.

For welders with incomplete exposure data, the individual respective exposure level was estimated using geometric mean exposure data obtained from welders working at the same workstation, engaged in similar tasks, or in the same company. The use of protection devices was then corrected for as described above. For two welders, to fill missing data for the exposure assessment, exposure data previously collected at the welding companies (11, 21) were also used. Only active welders (i.e., not retired or welders with non-welding work tasks) had either measured or assessed respirable dust data. In the end, 56 welders had respirable dust data at both timepoints (timepoint 1: measured *n* = 28, estimated *n* = 28; timepoint 2: measured *n* = 46, and estimated *n* = 10).

For the controls at timepoint 1, full-shift personal breathing zone samples of respirable dust were collected from two companies for 19 control subjects. From four companies, area-level air pollution monitoring of respirable dust was performed using a direct reading monitor, SidePak Model AM510 (TSI Incorporated) with a Dorr-Oliver cyclone (21). At timepoint 2, stationary area monitoring of respirable dust fractions was performed using a DustTrak DRX monitor (TSI Incorporated). At both timepoints, these monitors were placed at breathing zone height in the area where workers spent the most time during their work shifts. On average, the monitoring of each control lasted approximately 4 h at each company's work site. In companies where workers spent time at two different workstations, measurements were taken in both areas, and a time-weighted average of the two sites was calculated.

### Cumulative Dose

For timepoint 1, the cumulative dose was estimated by multiplying the value for respirable dust at timepoint 1 (adjusted for respiratory protection) with number of welding years at timepoint 1. Cumulative dose for timepoint 2 was calculated by multiplying respirable dust (adjusted for respiratory protection) at timepoint 2 with welding years at time point 2 and adding the resulting product to the cumulative dose for timepoint 1.

$$\begin{array}{r} Cumulative\;dose\;timepoint\text{\_}1\\ =Respirable\;dust\;timepoint\text{\_}1\times Years\;welding\;timepoint\text{\_}1\\ Cumulative\;dose\;timepoint\text{\_}2\\ =Cumulative\;dose\;timepoint\text{\_}1+[Respirable\;dust\;timepoint\text{\_}2\\ \times (Years\;welding\;timepoint\text{\_}2-Years\;welding\;timepoint\text{\_}1)] \end{array}$$

### Analysis of Metals on Respirable Dust Filters

Filters from timepoint 2 (*n* = 104 in total, out of which *n* = 100 are included in this study) were analyzed for element concentrations including Mn, and iron (24). After weighing, the filters were digested in 1 mL of concentrated nitric acid at 70°C for 16 h. After dilution with Milli-Q water, the metal concentrations were determined by inductively coupled plasma-mass spectrometry (ICP-MS; iCAP Q, Thermo Scientific GmbH, Germany) in collision cell mode, with kinetic energy discrimination, using helium as the collision gas. Detailed information on the metal content and the quality control of the analysis is included in Supplementary Table 1.

### Protein Measurement

Using the Proximity Extension Assay (PEA) technology (Olink Proteomics, Uppsala, Sweden) the serum samples were analyzed for 92 unique proteins related to neurobiological processes and neurological diseases (Neurology panel). The selected proteins have the following gene ontology terms: axon development (*n* = 17), axon guidance (*n* = 12), cell adhesion (*n* = 36). cell death (*n* = 21), cell differentiation (*n* = 37), cell growth (*n* = 13), cell metabolic process (*n* = 42), immune response (*n* = 22), MAPK cascade (*n* = 15), neurogenesis (*n* = 26), proteolysis (*n* = 8), signal transduction (*n* = 48), synapse assembly (*n* = 6), and other gene ontology terms (*n* = 11). One μL serum sample was used for the analysis. Processing, quality control as well as normalization were performed as previously described (25). Protein levels were reported as normalized protein expression (NPX) values on a log2-scale. The cut-offs for intra- or inter-assay CVs were <20% (calculated on linear data). In addition, proteins with 10% or more of the samples ≤ limit of detection were excluded from the analysis. Overall, out of the 92 proteins, 5 proteins (BDNF, GDNF, HAGH, BMP4, PLXNB1) were excluded due to low quality of the protein analysis; the statistical analyses were performed on 87 proteins only. The quality control of individual samples (pass/fail) was based on specific incubation and detection controls.

### Statistics

All statistical analyses were completed by using R statistical software version 3.4.4 (R Foundation for Statistical Computing. Vienna. Austria).

#### Evaluation of Differences Between the Study Groups

Characteristics are presented as median and 5–95 percentile for the continuous variables and percentage for categorical variables. Differences between groups were evaluated with the Kruskal–Wallis rank sum test (followed by Dunn's *post-hoc* test) (when comparing three groups), paired samples Wilcoxon test (when comparing two groups) for continuous variables, and Fisher's exact test for categorical variables.

#### Data Exploration Using Principal Component Analysis

PCA heatmaps were constructed using the *prince.plot* function in the swamp package in R. The function generates principal components that explain part of the variation in the protein data set and then tests each variable against these components to evaluate possible associations. Heatmaps depict *p*-values (–log10-transformed) of these associations. Hierarchical clustering of the variables was generated using the *hclust* function.

#### Evaluation of Differentially Expressed Proteins

Longitudinal analysis was performed using linear mixed models and associations between occupational groups (welder and control) with serum proteins were fitted using the *lmer* function in the lme4 package in R. Variance explained by fixed factors (${R^{2}}_{\text{m}}$) was calculated using *RsqGLM* function from the R package MuMin. The adjusted intraclass correlation coefficient was calculated using the *icc* function. The mixed models included study participants as random factors (random intercepts) and occupational group, age, and body-mass index (BMI) as fixed factors. Models were also constructed using additional variables such as physical activity, consumption of fish and vegetables, use of snus, and alcohol intake as random factors. Sensitivity analysis was performed on non-smokers only (*n* = 236).

Similar analysis using linear mixed model analysis was performed in welders after replacing the occupational group variable with respirable dust (in mg/m^3^, adjusted for respiratory protection), years of welding (in years), or cumulative exposure. In this case, the linear mixed models included study participants as random factors (random intercepts) and age, BMI and respirable dust, years of welding, or cumulative exposure as fixed factors. To note that age and welding years are correlated (*r*_S_ = 0.52 for all welders included in this study, *n* = 214).

For the analysis of the cross-sectional group, we used multivariable-adjusted linear models (adjusted for age and BMI) to evaluate the associations between occupational groups (welder and controls) as well as measures of exposure (welding years, respirable dust, cumulative exposure) in welders with serum proteins.

## Results

### Characteristics of the Study Participants

All study participants had a relatively healthy lifestyle: the majority were non-smokers, had low alcohol consumption and a medium-high intake of vegetables (Table 1). Compared with controls, welders were more likely to be born outside Sweden (*p* < 0.005) and to live in towns or countryside rather than in large or small cities (*p* < 0.005). There were no other significant differences between welders and controls. Age was significantly different between the study groups (*p* < 0.001); age for the cross-sectional group (one measurement) was more similar to the age for the longitudinal cohort (two measurements) at timepoint 1. There were no other significant differences between the longitudinal cohort and the cross-sectional group. BMI increased significantly at timepoint 2 compared with timepoint 1 in both welders and controls (Table 1). None of the other characteristics of the study participants changed significantly between the two timepoints.

**Table 1:** Characteristics of the longitudinal study group (cohort measured twice) and cross-sectional group (measured once) of welders and controls.

	**Cohort timepoint 1**		**Cohort timepoint 2**		**Cross-sectional group**		***p*-value^**i**^**	***p*-value^**°**^**
	**Welders (*n* = 56)**	**Controls (*n* = 67)**	**Welders (*n* = 56)**	**Controls (*n* = 67)**	**Welders (*n* = 102)**	**Controls (*n* = 89)**		
**Continuous variables - median (5–95 percentile)**								
Age (years)	44 (23–60)	44 (24–56)	50 (29–66)	50 (30–63)	44 (26–60)	44 (26–59)	-	-
Years welding	10 (1–28)	0 (0–12)	15 (4–34)	0 (0–12)	8 (2–29)	0 (0–4)	<0.001	-
Respirable dust (mg/m^3^)^a^	1 (0.3–4.2)	-	0.5 (0.1–4.3)	-	1.6 (0.1–6.8)	-	0.030	
Respirable dust adjusted (mg/m^3^)^b^	0.6 (0.2–4.2)	-	0.5 (0.1–1.8)	-	0.8 (0.1–3.4)	-	0.011	-
Cumulative exposure^c^	4.6 (0.4–35.3)	–	9.7 (1.9–36.8)	-	6.3 (0.7–33.6)	-	<0.001	-
Body-mass index (kg/m^2^)	27.2 (21.9–31.4)	27.5 (22.3–34.4)	28.1 (22.4–33.2)	28.1 (22.0–35.6)	28.7 (23.3–37.6)	27.5 (23.1–32.3)	<0.001	0.019
**Categorical variables**^**d**^ - *n* (%)^e^								
Country of birth (Sweden)	42 (75)	62 (93)	42 (75)	62 (93)	67 (66)	80 (91)	-	-
Education (university or higher)	2 (4)	7 (10)	2 (4)	9 (14)	9 (9)	10 (11)	0.946^j^	0.826^j^
Residence (large and small cities)^f^	11 (20)	34 (51)	10 (18)	29 (44)	22 (22)	43 (48)	0.956	0.611
Hobby exposure to particles^g^	13 (23)	11 (16)	12 (10)	14 (10)	29 (29)	14 (16)	1	0.513
Smoking history (ever smoked)	25 (45)	25 (37)	25 (45)	28 (42)	47 (47)	29 (33)	1	0.724
Smoking status (currently)								
Non-smoker	54 (96)	64 (96)	55 (98)	63 (95)	90 (89)	87 (98)	1	1
Party smoker	2 (4)	3 (4)	1 (2)	2 (3)	8 (8)	1 (1)		
Smoker	0 (0)	0 (0)	0 (0)	1 (2)	3 (3)	1 (1)		
Current snus use	16 (29)	12 (18)	16 (29)	11 (17)	32 (32)	21 (24)	1	1
Alcohol intake (≥3 times/week)	2 (4)	1 (1)	2 (4)	2 (3)	2 (2)	5 (6)	0.713^k^	0.714^k^
Vegetable intake (≥5 times/week)	36 (64)	43 (65)	32 (57)	50 (75)	59 (59)	57 (64)	0.684^l^	0.512^l^
Fish intake (at least once/week)	31 (55)	32 (48)	33 (59)	32 (48)	51 (50)	40 (45)	0.936^m^	0.427^m^
Physical activity (moderate/high)^h^	10 (18)	27 (40)	29 (52)	33 (50)	42 (42)	39 (44)	0.544^n^	0.715^n^

a *Measured by personal sampling or estimated*;

b *adjusted for personal respiratory protection equipment*;

c *cumulative exposure was calculated from adjusted respirable dust data and reported welding year experience*;

d *variables were categorized by “yes” and “no” unless otherwise stated*;

e *percentage calculated relative to the total valid answers*;

f *large and small cities as compared with towns and countryside*;

g *exposure to welding fumes, dust, engine exhaust. or engine diesel during leisure activities*;

h *physical activity that involves sweating at least once a week and for at least 30 min*;

i *p-value for the differences between welders timepoint 1 and welders timepoint 2 calculated using paired samples Wilcoxon test for continuous variables and Fisher's exact test for categorical variables*;

j *statistical test based on 5 categories for education from secondary school to university studies*;

k *statistical test based on 6 categories for intake of alcohol from every day to never*;

l *statistical test based on 8 categories from 3 per day or more to never*;

m *statistical test based on 7 categories from once per day or more to never*;

n *statistical test based on 4 categories from sedentary to intensive physical activity*;

° *p-value for the differences between controls timepoint 1 and controls timepoint 2 calculated using paired samples Wilcoxon test for continuous variables and Fisher's exact test for categorical variables*.

As expected, both years of welding and cumulative exposure increased significantly for welders at timepoint 2 compared with timepoint 1 (Table 1). Median respirable dust in air, with and without adjustment for respiratory protection, was significantly higher at timepoint 1 (0.6 and 1.0 mg/m^3^, respectively) compared with timepoint 2 (0.5 and 0.5 mg/m^3^, respectively). The median stationary *area*-*level* of respirable dust concentrations in the control companies were 0.09 mg/m^3^ (min-max: 0.02–0.2) for timepoint 1 and 0.03 mg/m^3^ (min-max: 0.02–0.06) at timepoint 2. The cross-sectional group had median respirable dust levels of 0.8 and 1.6 mg/m^3^, with and without adjustment for respiratory protection, respectively, which was higher than in the longitudinal cohort. Mn concentrations were measured in the respirable dust at timepoint 2 and the median concentration was 0.085 mg/m^3^ (5–95 percentile: 0.003, 0.584 mg/m^3^). The median adjusted respirable Mn concentration was 0.049 mg/m^3^ (5–95 percentile: 0.003, 0.314 mg/m^3^). Of those welders who worked unprotected (*n* = 59), 26 were exposed to levels of Mn dust above the OEL (0.05 mg/m^3^) at timepoint 2. The correlation between Mn and respirable dust in air was *r*_S_ = 0.90 and the correlation after adjusting for respiratory protection was *r*_S_ = 0.88 (data at timepoint 2 evaluated, *n* = 100).

### Variation of Neurology-Related Proteins in Serum

**Figure 2:**
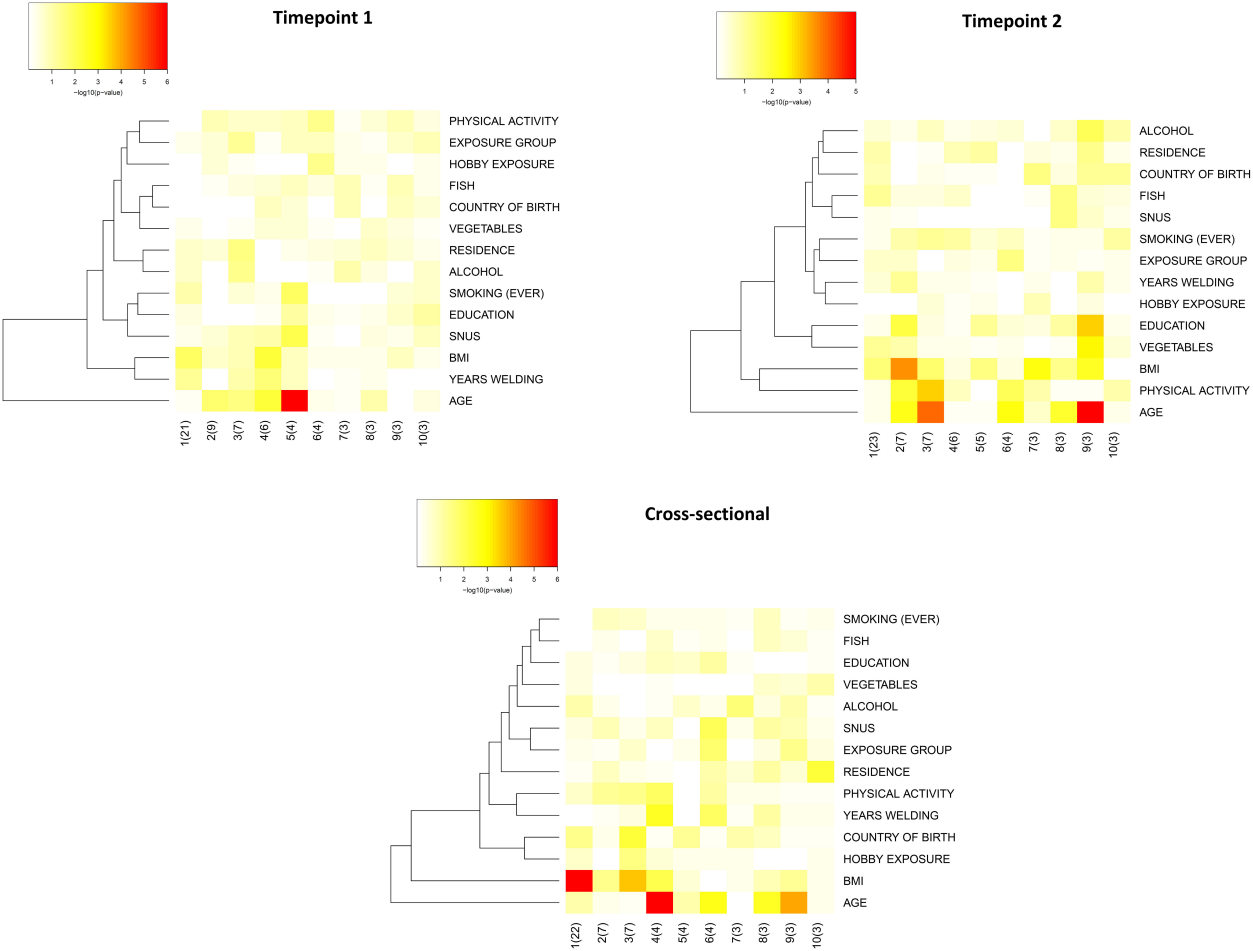
Heatmaps of the principal components (PC) that explain the variation in the study groups. Heatmaps were constructed using input data from linear regression of association between the principal components of the data and the biological annotations. The influence of the biological annotations on the overall variation is plotted in a heatmap based on the *p*-value of the association. Input data was the normalized protein expression values (on a log2-scale). “group” refers to occupational group i.e., welders and controls; “residence” refers to current residence in large and small cities as compared with towns and countryside; “hobby exposure” exposure to welding fumes, dust, engine exhaust or engine diesel during leisure activities; “country of birth” is categorized as Sweden or outside Sweden; “education” is assigned to 5 categories for education from secondary school to university studies; “vegetables” frequency of intake of vegetables and is assigned to 8 categories from 3 per day or more to never; “fish” frequency is based on 7 categories from once per day or more to never; “physical activity” is based on 4 categories from sedentary to intensive physical activity; “ever smoking” stands for current or previous smoking and is categorized as “yes” and “no”; “alcohol” stands for frequency of alcohol intake and is stratified in 6 categories from every day to never.

Principal component analysis (PCA) was performed to evaluate to which extent characteristics of the study participants could explain the variation in serum levels of neurology-related proteins. The complete description of the 87 measured proteins is appended in Supplementary Table 2. Cohort timepoints 1 and 2, and the cross-sectional group, showed similar patterns in the PCA heatmap with age and BMI being the most significant parameters explaining protein variation (Figure 2). For this reason, age and BMI were used as covariates in the statistical analyses to evaluate differential protein expression in relation to welding (see below).

**Figure 3:**
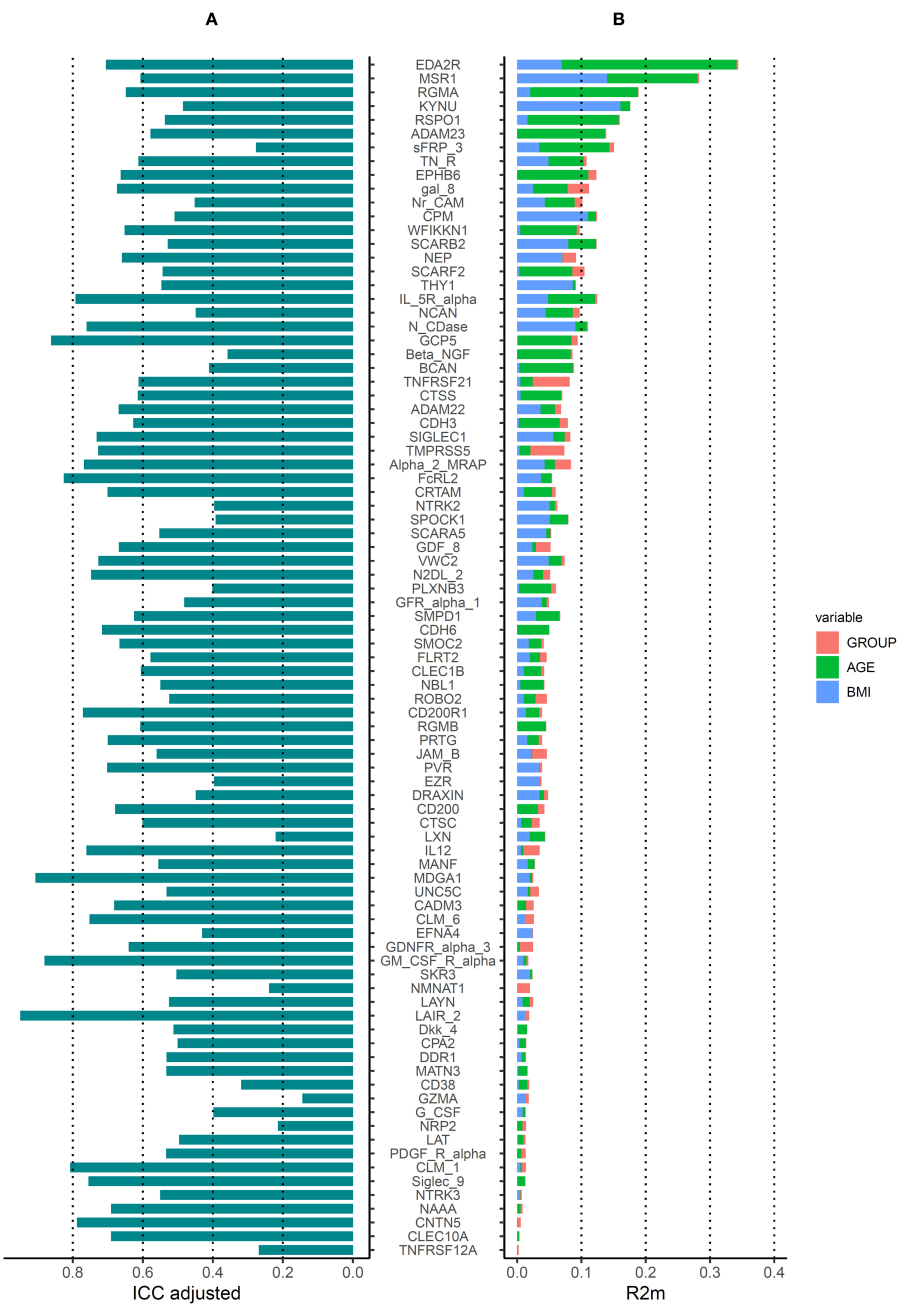
Variation of serum protein from the Olink neurology panel. **(A)** Intraclass correlation coefficients (ICCs) estimated in linear mixed models including occupational group, age, BMI as fixed factors, and individual as random factors. **(B)** Protein variance explained ($R_{\text{m}}^{2}$) by occupational group, age and BMI generated from models including occupational group, age or BMI as fixed factors (one by one) and individual as random factors. Proteins are ordered according to the $R_{\text{m}}^{2}$ of a linear mixed model including occupational group, age and BMI as fixed factors and individual as random factors.

In addition, we evaluated the intra-class coefficient (ICC) of linear mixed models where individuals were random factors and occupational group, age and BMI were fixed factors, and evaluated the contribution of each of these fixed factors to the protein variance. Approximately half of the proteins (*n* = 45) had a relatively low intra-individual variation (i.e., ICC > 0.6) out of which 6 had a low intra-individual variation (i.e., ICC > 0.8) (Figure 3A). The protein variance (${R^{2}}_{\text{m}}$) explained by occupational group (welders or controls), age and BMI, is presented in Figure 3B. For 17 proteins, age explained more than 5% of the variation (5–27%) while for 11 proteins BMI explained more than 5% of the variation (5–16%). Occupational group explained more than 5% of the variation for 2 proteins TNFRSF21 (6%) and TMPRSS5 (5%). The proportion of protein variance explained by occupational group, age, and BMI together in a combined model varied between 1 and 30% for most of the proteins (for 5 proteins ${R^{2}}_{\text{m}}<$ 1%). The top 5 proteins most influenced by age were EDA2R, RGMA, RSPO1, MSR1, and ADAM23 (${R^{2}}_{\text{m}}$ 27–14%) while the top 5 proteins most influenced by BMI were KYNU, MSR1, CPM, N_CDase, and THY1 (${R^{2}}_{\text{m}}$ 16–9%).

The addition of extra fixed factors to the models (e.g., physical activity, consumption of fish and vegetables, use of snus and alcohol intake) did not have major impact on the ICC or ${R^{2}}_{\text{m}}$ (ICC of the combined model >0.6 for 42 proteins, ${R^{2}}_{\text{m}}$ of the combined model between 2 and 32%) (Supplementary Figure 1) compared to models only considering occupational group, age, and BMI.

### Differential Protein Expression in Relation to Welding

Using a longitudinal analysis (linear mixed models) we identified 5 out of 87 serum proteins that were differently expressed in welders compared with controls (TNFRSF21, TMPRSS5, NEP, GDF8, and NMNAT1, Table 2, *p* < 0.05). None of the associations were significant after correcting for multiple testing. Sensitivity analysis performed in non-smokers at both timepoints, resulted in very similar effect estimates as the main analysis. In addition, IL12 and gal_8 also reached the significance threshold (*p*-value 0.045 and 0.040, respectively).

**Table 2:** Differentially expressed proteins in serum between welders and controls in the longitudinal study group (linear mixed models) and corresponding data for the cross-sectional group (linear models).

**Protein**	**Linear Mixed Models (*****n*** **=** **246)**			**Linear models (cross-sectional group) (*****n*** **=** **191)**		
	**$R_{\text{m}}^{2}$ (%)^**a**^**	**Beta (SE)^**b**^**	**p^**c**^**	***R*^**2**^ (%)^**d**^**	**Beta (SE)^**e**^**	**p^**f**^**
TNFRSF21	8	−0.112 (0.039)	0.004	4	−0.008 (0.037)	0.838
TMPRSS5	7	−0.171 (0.059)	0.004	−1	−0.033 (0.056)	0.554
NEP	10	0.257 (0.116)	0.027	10	−0.022 (0.102)	0.833
GDF8	6	0.185 (0.087)	0.033	5	0.066 (0.072)	0.365
NMNAT1	2	0.256 (0.126)	0.043	7	0.28 (0.129)	0.032

*SE, standard error*;

a *Variance explained by fixed factors (group, age, body-mass index)*;

b *regression coefficient from linear mixed models interpreted as standard deviation difference in protein levels compared to controls, adjusted for age, body-mass index variables as fixed factors, and participant as random factors*;

c P-value from test of contribution of group inclusion (welders and controls) to protein variance using an analysis of variance approach with Satterthwaite approximation for degrees of freedom (Bonferroni-adjusted threshold for the p-value: 0.05/87 = 5.7*10^−4^);

d *variance in protein levels explained by the linear model*;

e *regression coefficient from multivariable-adjusted linear models interpreted as standard deviation difference in protein levels compared to controls adjusted for age, body-mass index*;

f *p-value from the linear model to test the difference between welders and control*.

One of the proteins, nicotinamide/nicotinic acid mononucleotide adenyltransferase 1 (NMNAT1) was also differently expressed in the cross-sectional group (Table 2). The magnitude and direction of effect was similar between the longitudinal and cross-sectional analyses. Complete output data from the differential expression analysis between welders and controls is appended in Supplementary Table 3. Gene ontology terms (biological process) for NMNAT1 are included in Supplementary Table 4.

In order to investigate dose-response relationships, we evaluated longitudinal associations (welders only) between serum proteins and measurements of exposure to welding, i.e., respirable dust (adjusted for respiratory protection, *n* = 42 welders with two measurements), years of welding (*n* = 56 welders), and cumulative exposure (*n* = 42) (Table 3).

**Table 3:** Differentially expressed proteins in serum in welders associated with exposure expressed as respirable dust (adjusted for personal respiratory protection equipment), years welding and cumulative exposure in the longitudinal study group (linear mixed models) and corresponding data for the cross-sectional group (linear models).

**Protein**	**Linear mixed models**			**Linear models (cross-sectional group)**		
	**$R_{\text{m}}^{2}$ (%)^**a**^**	**Beta (SE)^**b**^**	***p*^**c**^**	***R*^**2**^ (%)^**d**^**	**Beta (SE)^**e**^**	***p*^**f**^**
**Respirable dust (*****n*** **=** **84/96)**						
KYNU	20	0.188 (0.063)	0.003	16	0.068 (0.046)	0.139
CTSC	12	0.099 (0.041)	0.016	8	0.045 (0.033)	0.172
GFRα1	12	0.061 (0.027)	0.027	10	−0.024 (0.021)	0.244
NBL1	10	0.034 (0.016)	0.036	2	0.025 (0.013)	0.071
**Years welding (*****n*** **=** **112/101)**						
GCSF	17	−0.025 (0.006)	<0.001^#^	7	−0.018 (0.008)	0.023
CRTAM	16	−0.022 (0.007)	0.002	1	−0.005 (0.007)	0.490
ADAM23	27	−0.023 (0.009)	0.011	−1	−0.009 (0.008)	0.227
IL12	9	−0.024 (0.01)	0.013	9	0.007 (0.008)	0.395
EFNA4	9	−0.008 (0.003)	0.014	22	−0.008 (0.003)	0.016
LAIR2	5	−0.033 (0.014)	0.017	1	−0.024 (0.016)	0.141
CTSS	17	−0.009 (0.004)	0.025	15	−0.006 (0.003)	0.042
CLM1	5	−0.017 (0.008)	0.028	4	−0.001 (0.008)	0.854
CLM6	6	−0.007 (0.003)	0.030	12	−0.007 (0.003)	0.033
VWC2	7	−0.013 (0.006)	0.047	25	−0.02 (0.006)	0.001
GFRα1	10	−0.009 (0.005)	0.049	12	−0.006 (0.004)	0.118
**Cumulative exposure (*****n*** **=** **84/95)**						
SCARB2	21	0.007 (0.002)	0.001	16	−0.001 (0.002)	0.635
LXN	14	0.004 (0.002)	0.008	4	0 (0.001)	0.820
PRTG	17	0.009 (0.003)	0.009	0	0 (0.002)	0.981
MDGA1	7	0.014 (0.006)	0.013	−1	−0.002 (0.004)	0.681
CD38	9	0.009 (0.004)	0.017	3	−0.002 (0.002)	0.243
GCP5	18	0.014 (0.006)	0.020	5	−0.002 (0.004)	0.621
FcRL2	16	0.011 (0.005)	0.024	1	0 (0.003)	0.988
sFRP_3	19	0.013 (0.007)	0.050	−2	−0.001 (0.003)	0.771

*SE, standard error*;

a *Variance explained by fixed factors (respirable dust/years welding/cumulative exposure, age, body-mass index)*;

b *regression coefficient from linear mixed models interpreted as standard deviation difference in protein levels per respirable dust unit increase/numbers of years welding/cumulative exposure unit increase, adjusted for age, body-mass index variables as fixed factors, and participant as random factors*;

c p-value from test of contribution of respirable dust/years welding/cumulative exposure to protein variance using an analysis of variance approach with Satterthwaite approximation for degrees of freedom (Bonferroni-adjusted threshold for the p-value: 0.05/87 = 5.7*10^−4^);

d *variance in protein levels explained by the linear model*;

e *regression coefficient from linear mixed models interpreted as standard deviation difference in protein levels per respirable dust unit increase/numbers of years welding/cumulative exposure unit increase, adjusted for age and body-mass index variables*;

f *p-value from the linear model to test the association with exposure variables (respirable dust/years welding/cumulative exposure)*;

# *significant after adjustment for multiple testing (Bonferroni)*.

None of the five proteins (TNFRSF21, TMPRSS5, NEP, GDF8, and NMNAT1), differentially expressed in welders vs. controls, were associated with any measurement of exposure to welding (Supplementary Table 5). The protein neuroblastoma 1, DAN family BMP antagonist (NBL1), was associated with respirable dust in the longitudinal analysis (*p* = 0.036) and non-significantly in the cross-sectional analysis (*p* = 0.071). We identified 5 proteins (GCSF, EFNA4, CTSS, CLM6, VWC2) significantly associated with years of welding both in the longitudinal and the cross-sectional analysis (Table 3). Gene ontology terms (biological process) related to these proteins are included in Supplementary Table 4. GCSF was the only protein significantly associated with years welding after multiple comparison adjustment (Table 3). None of the other proteins were associated with respirable dust or cumulative exposure in the cross-sectional analyses.

### Differential Protein Expression in Relation to Other Factors

Finally, using the same statistical approach we evaluated the associations of serum proteins with age and BMI. These two variables were indicated by the PCA analysis to explain part of the variation in the serum proteins. Our results suggest that 38 serum proteins (18 after multiple comparison adjustment) were associated with age in linear mixed models adjusted for BMI (Supplementary Table 6). In addition, 19 serum proteins were also associated with age in the cross-sectional group (Supplementary Table 4). We identified 41 proteins (11 proteins after multiple comparison adjustment) that were associated with BMI in linear mixed models adjusted for age (Supplementary Table 7) of which 29 were significant in the cross-sectional group (Supplementary Table 7).

## Discussion

In this exploratory study, we investigated the influence of welding on levels of putative neurology-related serum proteins as a proxy of exposure-related changes in the nervous system. We found that welders exposed to respirable dust concentrations below the current Swedish OEL (2.5 mg/m^3^) show change in serum levels of proteins related to neurological processes and neurologic disease. We do not know if the observed protein changes are related to exposure to Mn, particles or other factors present in the occupational environment of welders as we did not observe dose-response associations with different measures of exposure to welding fumes. In addition, we identified several proteins that are associated with age and BMI.

We found that being a welder was associated with higher serum levels of NMNAT1 in both longitudinal and cross-sectional analyses. NAD+/NADH are key players in redox reactions providing energy for metabolic reactions. Nicotinamide mononucleotide is converted into NAD+ by NMNAT that exists in 3 different isoforms. NMNAT1 is the nuclear form (26) and is widely expressed in all tissues, including the brain (www.proteinatlas.org). Further, NMNAT1 has a general role in neuronal maintenance, it prevents axon degeneration and has been shown to protect neurons from reactive oxygen species (26–28). Increased levels of NMNAT1 in welders may therefore reflect a protective response mechanism against the effects of long-term neurotoxic exposures, such as from oxidative stress (29) which has been associated with exposure to welding fumes (30). Studies in a mice model of Alzheimer disease have linked NMNAT1 to protection against Alzheimer disease (31, 32). There are, to our knowledge, no reports linking exposure to welding fumes to Alzheimer disease, and this needs to be explored in future studies. We would like to note that NMNAT1 is also associated with retinal function (33) and it is possible that the association found in our study could be related to exposure to UV radiation during welding. Exposure to UV radiation during welding has been classified as carcinogenic to humans (34).

In the dose-response analysis among welders only, associations with other serum proteins were found. Respirable dust was associated with increased expression of NBL1 (although not significant in the cross-sectional analysis), a protein widely expressed in the body and in blood expressed in T-cells (proteinatlas.org). NBL1 was initially identified as a tumor suppressor in a neuroblastoma cell line (35) and was later found important for nervous system and bone development (36). There is hitherto no relation described with NBL1 and neurodegenerative diseases or with manganese or iron. Together with the fact that the association with respirable dust was weak in the cross-sectional group, suggest that the findings for NBL1 should be interpreted cautiously.

In the dose-response analysis among welders only, we identified 5 proteins (GCSF, EFNA4, CTSS, CLM6, VWC2) that were associated with welding years, both in longitudinal and cross-sectional analyses. The respective proteins were negatively associated with welding years and apart from CTSS they were not associated with age. GCSF (granulocyte-colony stimulating factor) is a growth factor that has neuroprotective effects (37) and was shown to be expressed in lower levels in the brain of individuals with neurodegenerative diseases such as Parkinson disease and the rare degenerative disease multiple system atrophy (38). EFNA4 (EphrinA4) belongs to the ephrin family of proteins responsible for axonal guidance (39) and is involved in modulation of neuronal regeneration after injury (40). CTSS (cathepsin S) is a protease particularly expressed in the microglial cells of the central nervous system and *in vivo* studies in mice indicated CTSS upregulated with age and neuroinflammation (41, 42). CLM6 (also known as CD300c) is a receptor found on the surface of immune cells with yet an unclear function. VWC2 (also known as the brorin) is expressed both in neural tissues in embryos and in neurons in the adult mouse brain and is involved in neurogenesis (43). In a recent study plasma EFNA4, CLM6, and VWC2 were negatively associated with general fluid cognitive ability in old individuals, an association that appeared to be mediated by brain volume (44).

Although the protein changes identified cannot be clearly linked to Mn exposure of the welders, it should be noted that the Mn levels in the welders were high in relation to the current Swedish OEL, i.e., 0.05 mg/m^3^. The median value of the adjusted respirable Mn concentrations was close to 0.05 mg/m^3^ and half of the welders were assessed to be exposed to Mn concentrations above the OEL. Further, it should be noted that the measured unadjusted respirable Mn concentrations did not decrease compared with measured unadjusted concentrations reported in 2003–2005, i.e., 0.08 mg/m^3^ (11). Thus, the respirable Mn exposure levels among welders in Sweden have not declined for the past 15 years although that the OEL has been reduced from 0.1 to 0.05 mg/m^3^.

Being exposed above the OEL of Mn could increase the risk of adverse health effects on the nervous system in welders, as suggested from our study, and further protective measures should be taken. It is important that the general mechanical ventilation at the workplace is efficient and that local exhaust ventilation is available and used to reduce emissions at the source. Exposure can further be efficiently reduced by use of e.g., powered air purifying respirators, especially those with double visors.

This is an exploratory study, but we performed a rather strict analysis to identify proteins related to welding, by evaluating the proteins both in a longitudinal cohort and a cross-sectional group. It should be noted that changes in protein levels in this study were measured in serum and the relevance for processes in the brain should be interpreted cautiously as we do not know to which degree serum levels reflect expression levels in more relevant tissues. Therefore, to evaluate their relevance, it would be useful to measure the same proteins, e.g., NMNAT1, GCSF in other welding cohorts or in experimental studies of welding fumes. Proteins that were associated with welding in this study, if validated, could potentially function as biomarkers of neurotoxic effects of exposure to welding fumes. This is a longitudinal study, however, we acknowledge the limitation of only having two sampling points. The limited number of timepoints could overlook dynamic changes both in protein expression and exposure to welding fumes. A weakness with the study is that Mn exposure was not assessed at both timepoints, although we had a strong correlation between Mn and respirable dust at timepoint 2. Mn exposure is difficult to estimate by measurements in biological samples, partly due to highly efficient homeostatic mechanisms, and there is no validated biomarker for Mn exposure.

All in all, our study indicates that low-to-moderate exposure to welding fumes is associated with consistent changes in circulating levels of neurology-related proteins, that might be indicators of an increased risk for future disease. This highlights the need to further reduce the exposure levels to welding fumes.

## Data Availability Statement

The raw data supporting the conclusions of this article will be made available by the authors, without undue reservation.

## Ethics Statement

The studies involving human participants were reviewed and approved by Regional Ethic Committee Lund University, Sweden. The patients/participants provided their written informed consent to participate in this study.

## Author Contributions

KW, MH, MA, and KB planned and designed the research and were involved in data interpretation. EA did the recruitment and medical examination of the study participants and data collection. MH did the exposure measurements. MH and TT did the exposure assessment. TL conducted the metal analysis. AG and TT conducted the statistical analysis. AG, KW, and KB wrote the manuscript. All authors commented on previous versions of the manuscript and read and approved the final manuscript.

## Conflict of Interest

The authors declare that the research was conducted in the absence of any commercial or financial relationships that could be construed as a potential conflict of interest.

## Supplementary Material

Supplementary Figure 1 Variation of serum protein from the Olink neurology panel. **(A)** Intraclass correlation coefficients (ICCs) estimated in linear mixed models including occupational group, age, BMI, physical activity, consumption of fish and vegetables, use of snus and alcohol intake as fixed factors, as well as individual as random factors. **(B)** Protein variance explained ($R_{\text{m}}^{2}$) by occupational group, age, and BMI generated from models including occupational group, age, BMI, physical activity, consumption of fish and vegetables, use of snus and alcohol intake as fixed factors (one by one) and individual as random factors. Proteins are ordered according to the $R_{\text{m}}^{2}$ of a linear mixed model including occupational group, age, BMI, physical activity, consumption of fish and vegetables, use of snus and alcohol intake as fixed factors as well as individual as random factors.

Supplementary Table 1 Metal concentrations in respirable dust measured among welders participating at timepoint 2.

Supplementary Table 2 Normalized protein expression values. Data presented as median and interquartile range.

Supplementary Table 3 Complete output of the differential protein expression analysis between welders and controls in the longitudinal study group (linear mixed models) and corresponding data for the cross-sectional group (linear models).

Supplementary Table 4 Gene ontologies (biological process category) related to proteins that were differentially expressed in both the longitudinal and the cross-sectional analyses (according to uniprot.org).

Supplementary Table 5 Associations of the significantly differentially expressed proteins in welders and controls with exposure to welding expressed as respirable dust (adjusted for personal respiratory protection equipment), years welding and cumulative exposure in welders only (linear mixed models).

Supplementary Table 6 Proteins associated with age in the longitudinal study group (linear mixed models) and corresponding data for the cross-sectional group (linear models).

Supplementary Table 7 Proteins associated with BMI in the longitudinal study group (linear mixed models) and corresponding data for the cross-sectional group (linear models).
