# Supplementary material for: Exposure to Mild Steel Welding and Changes in Serum Proteins With Putative Neurological Function—A Longitudinal Study
Source: Front Public Health. 2020 Aug 28;8:422. doi: 10.3389/fpubh.2020.00422 (PMC7485227; doi:10.3389/fpubh.2020.00422)
Supplement: Supplementary Table 1 — Metal concentrations in respirable dust measured among welders participating at timepoint 2. [file Table_1.pdf]

Supplementary Table 1. Metal concentrations in respirable dust measured among welders participating at timepoint 2.

Metal **(mg/m^3)**	***Median***	***Minimum***	***Maximum***
Aluminium	0.0027	0.0001	0.0468
Cadmium	0.0000	0.0000	0.0000
Cobalt	0.0000	0.0000	0.0006
Chromium	0.0004	0.0000	0.0339
Copper	0.0032	0.0001	0.0516
Iron	0.5515	0.0111	5.9907
Potassium	0.0122	-0.0096	0.8609
Magnesium	0.0006	0.0000	0.3570
Manganese	0.0896	0.0009	1.5350
Nickel	0.0005	0.0000	0.0151
Lead	0.0002	0.0000	0.0021
Thorium	0.0000	0.0000	0.0000
Vanadium	0.0000	0.0000	0.0009
Zinc	0.0022	0.0002	0.0574

Metal values are based on all samples measured at timepoint 2 (n=104). The number of welders included in the analysis of associations with neurology-protein were n=100 and the metal concentrations therefore slightly differ for Mn and Fe as reported in the main text. The detection limits were calculated as three times the standard deviation [SD] of blank filters and are as follows: 0.005 µg (Co, Cd, Tl, Pb, Th), 0.007 µg (V, Ni), 0.01 µg (Cu), 0.02 µg (Mn), 0.05 µg (Cr, Zn), 0.08 µg (Mg), 0.15 µg (Al), 0.89 µg (Fe), and 7.4 µg (K). Analytical accuracy was verified using certified reference filters in the analysis of samples, n = 20 (Trace Metal on Filter Media D, Part nr. QC-TMFM-D, Lot 1530803; High-Purity Standards, USA). The obtained (mean ± SD) vs. certified values were 2.46 ± 0.03 µg, 2.45 ± 0.04, 2.51 ± 0.04, 2.49 ± 0.03, 2.51± 0.03 vs. 2.50 ± 0.03 for V, Cr, Co, Ni, and Cu, respectively; 2.47 ± 0.04 and 2.45 ± 0.03 vs. 2.50 ± 0.05 for Tl and Pb, respectively; 1.04 ± 0.02 and 0.98 ± 0.01 vs. 1.00 ± 0.01 for Mn and Cd, respectively; 2.70 ± 0.21 vs. 2.50 ± 0.1 for Fe; 2.39 ± 0.07 vs. 2.50 ± 0.2 for Zn; and 49.0 ± 0.86 vs. 50.0 ± 0.5 for Al. No reference values were available for Mg, K, or Th.
