# Supplementary material for: Exposure to Mild Steel Welding and Changes in Serum Proteins With Putative Neurological Function—A Longitudinal Study
Source: Front Public Health. 2020 Aug 28;8:422. doi: 10.3389/fpubh.2020.00422 (PMC7485227; doi:10.3389/fpubh.2020.00422)
Supplement: Supplementary Table 2 — Normalized protein expression values. Data presented as median and interquartile range. [file Table_2.pdf]

Supplementary table 2. Normalized protein expression values. Data presented as median and interquartile range.

Olink symbol	Cohort Timepoint 1		Cohort Timepoint 2		Cross-sectional group	
	Controls	Welders	Controls	Welders	Controls	Welders
ADAM22	3.88 (3.57-4.22)	3.82 (3.57-4.05)	3.81 (3.60-4.06)	3.78 (3.54-3.96)	3.84 (3.59-4.03)	3.86 (3.61-4.13)
ADAM23	2.65 (2.29-3.06)	2.65 (2.31-3.07)	2.54 (2.26-2.80)	2.44 (1.83-2.77)	2.60 (2.35-3.01)	2.56 (2.22-2.92)
Alpha_2_MRAP	7.49 (7.32-7.87)	7.47 (7.27-7.64)	7.55 (7.38-7.83)	7.53 (7.35-7.65)	7.49 (7.32-7.74)	7.51 (7.29-7.70)
BCAN	4.90 (4.74-5.15)	4.91 (4.64-5.11)	4.97 (4.69-5.21)	4.99 (4.65-5.20)	4.98 (4.70-5.26)	5.00 (4.79-5.21)
Beta_NGF	0.91 (0.81-1.06)	0.87 (0.75-1.02)	0.82 (0.67-0.98)	0.83 (0.73-0.98)	0.85 (0.73-1.07)	0.84 (0.65-1.00)
CADM3	1.31 (0.98-1.57)	1.28 (1.00-1.51)	1.41 (1.00-1.66)	1.34 (0.94-1.57)	1.32 (0.97-1.68)	1.32 (1.16-1.57)
CD200	5.06 (4.88-5.27)	4.99 (4.85-5.18)	4.97 (4.84-5.22)	4.97 (4.76-5.15)	5.09 (4.85-5.24)	5.04 (4.86-5.21)
CD200R1	4.26 (4.01-4.43)	4.22 (3.97-4.37)	4.24 (4.03-4.43)	4.19 (3.99-4.40)	4.28 (4.11-4.48)	4.32 (4.04-4.58)
CD38	3.88 (3.71-4.08)	3.80 (3.56-4.09)	4.17 (3.94-4.44)	4.20 (3.99-4.44)	4.04 (3.76-4.33)	4.15 (3.91-4.36)
CDH3	5.97 (5.74-6.23)	5.97 (5.72-6.10)	5.95 (5.74-6.31)	5.96 (5.66-6.15)	6.00 (5.83-6.18)	5.99 (5.76-6.26)
CDH6	3.92 (3.73-4.14)	3.91 (3.79-4.06)	3.89 (3.72-4.04)	3.86 (3.71-4.04)	3.89 (3.71-4.04)	3.94 (3.76-4.06)
CLEC10A	3.22 (2.83-3.56)	3.17 (2.99-3.52)	3.25 (3.05-3.48)	3.18 (2.87-3.56)	3.08 (2.83-3.40)	3.20 (2.95-3.47)
CLEC1B	10.72 (10.50-10.92)	10.75 (10.47-11.05)	10.52 (10.24-10.81)	10.55 (10.32-10.75)	10.52 (10.32-10.75)	10.59 (10.27-10.90)
CLM_1	4.36 (4.06-4.57)	4.32 (3.92-4.63)	4.33 (3.95-4.58)	4.21 (3.86-4.53)	4.24 (3.82-4.57)	4.24 (3.85-4.53)
CLM_6	5.17 (5.04-5.33)	5.12 (4.97-5.26)	5.13 (4.98-5.30)	5.09 (4.93-5.22)	5.11 (4.96-5.24)	5.12 (4.97-5.32)
CNTN5	5.42 (5.17-5.69)	5.48 (5.20-5.75)	5.33 (5.16-5.57)	5.52 (5.20-5.69)	5.42 (5.10-5.72)	5.45 (5.11-5.71)
CPA2	9.32 (9.00-9.73)	9.40 (9.01-9.67)	9.50 (9.20-9.85)	9.47 (9.00-9.81)	9.62 (9.18-9.95)	9.60 (9.04-9.99)
CPM	6.43 (6.26-6.63)	6.43 (6.26-6.53)	6.42 (6.24-6.58)	6.40 (6.25-6.51)	6.43 (6.14-6.60)	6.44 (6.24-6.57)
CRTAM	4.05 (3.58-4.35)	4.09 (3.86-4.42)	3.74 (3.50-4.13)	3.86 (3.53-4.16)	3.95 (3.64-4.33)	4.09 (3.74-4.39)
CTSC	1.85 (1.65-2.14)	2.00 (1.76-2.22)	1.65 (1.43-1.86)	1.79 (1.61-1.94)	1.80 (1.55-2.13)	1.72 (1.51-1.95)
CTSS	5.28 (5.14-5.38)	5.25 (5.09-5.46)	5.13 (5.03-5.32)	5.21 (5.05-5.29)	5.23 (5.08-5.45)	5.14 (4.99-5.34)
DDR1	5.55 (5.43-5.76)	5.63 (5.48-5.82)	5.59 (5.42-5.77)	5.56 (5.49-5.72)	5.58 (5.43-5.74)	5.58 (5.45-5.71)
Dkk_4	2.12 (1.93-2.34)	2.21 (1.91-2.42)	2.06 (1.92-2.19)	2.10 (1.88-2.31)	2.11 (1.91-2.29)	2.06 (1.84-2.29)
DRAXIN	2.43 (2.10-2.74)	2.41 (2.14-2.74)	2.55 (2.38-2.93)	2.52 (2.38-2.75)	2.57 (2.29-2.86)	2.52 (2.30-2.81)
EDA2R	3.30 (3.02-3.58)	3.26 (2.97-3.57)	3.53 (3.16-3.73)	3.40 (3.17-3.70)	3.29 (3.01-3.51)	3.27 (3.00-3.60)
EFNA4	2.91 (2.82-3.06)	2.94 (2.76-3.05)	2.91 (2.80-3.06)	2.90 (2.80-3.04)	2.90 (2.72-3.06)	2.93 (2.77-3.11)
EPHB6	2.82 (2.61-3.05)	2.81 (2.64-2.88)	2.79 (2.54-2.95)	2.73 (2.52-2.93)	2.79 (2.55-3.01)	2.82 (2.60-2.99)
EZR	4.71 (4.56-4.88)	4.73 (4.60-4.94)	4.72 (4.55-4.85)	4.72 (4.57-4.87)	4.65 (4.51-4.85)	4.79 (4.65-4.97)
FcRL2	4.40 (4.15-4.69)	4.48 (4.10-4.81)	4.37 (4.16-4.72)	4.44 (4.02-4.73)	4.53 (4.18-4.72)	4.48 (4.16-4.82)
FLRT2	1.21 (1.01-1.36)	1.19 (1.00-1.35)	1.14 (0.98-1.29)	1.05 (0.92-1.29)	1.11 (0.94-1.33)	1.19 (0.98-1.35)
G_CSF	1.63 (1.44-1.90)	1.66 (1.50-1.98)	1.66 (1.47-1.89)	1.65 (1.37-1.95)	1.69 (1.46-1.92)	1.77 (1.44-1.95)
gal_8	5.80 (5.55-6.05)	5.73 (5.48-5.90)	5.72 (5.50-5.96)	5.62 (5.28-5.84)	5.70 (5.40-5.96)	5.65 (5.38-5.84)
GCP5	4.12 (3.68-4.54)	4.07 (3.56-4.32)	4.00 (3.56-4.35)	3.88 (3.43-4.38)	4.07 (3.61-4.51)	3.85 (3.50-4.30)
GDF_8	3.68 (3.30-4.12)	3.84 (3.37-4.32)	3.74 (3.29-4.10)	3.87 (3.47-4.32)	3.75 (3.43-4.13)	3.82 (3.50-4.20)
GDNFR_alpha_3	3.68 (3.56-3.92)	3.64 (3.50-3.84)	3.67 (3.56-3.89)	3.65 (3.44-3.87)	3.66 (3.48-3.86)	3.59 (3.44-3.80)
GFR_alpha_1	5.76 (5.56-5.90)	5.72 (5.56-5.94)	5.70 (5.53-5.84)	5.67 (5.50-5.82)	5.68 (5.49-5.88)	5.69 (5.56-5.86)
GM_CSF_R_alpha	5.62 (5.08-6.01)	5.61 (5.08-5.96)	5.49 (5.03-6.07)	5.52 (5.08-5.86)	5.46 (4.62-5.88)	5.59 (5.03-5.99)
GZMA	4.01 (3.73-4.26)	4.03 (3.81-4.17)	3.95 (3.63-4.21)	3.91 (3.71-4.12)	3.90 (3.69-4.19)	4.00 (3.70-4.20)
IL_5R_alpha	3.21 (2.71-3.74)	3.21 (2.94-3.60)	3.19 (2.89-3.82)	3.22 (3.03-3.56)	3.26 (2.81-3.74)	3.19 (2.78-3.61)
IL12	7.62 (7.29-7.94)	7.53 (6.85-7.84)	7.64 (7.26-8.00)	7.37 (6.96-7.75)	7.63 (7.17-8.02)	7.55 (7.24-8.02)
JAM_B	7.57 (7.29-7.71)	7.41 (7.15-7.59)	7.49 (7.34-7.76)	7.42 (7.28-7.66)	7.43 (7.18-7.65)	7.49 (7.31-7.67)
KYNU	6.28 (5.98-6.68)	6.30 (5.85-6.78)	6.38 (6.05-6.71)	6.30 (6.08-6.63)	6.31 (5.97-6.84)	6.46 (6.09-6.89)
LAIR_2	3.34 (2.87-3.83)	3.33 (2.75-3.91)	3.38 (2.91-3.88)	3.34 (2.77-3.83)	3.34 (2.92-3.81)	3.58 (2.93-3.96)
LAT	3.41 (2.98-3.76)	3.46 (3.13-3.69)	3.34 (3.07-3.61)	3.26 (2.99-3.45)	3.25 (3.05-3.60)	3.25 (2.96-3.48)
LAYN	4.22 (4.02-4.48)	4.15 (4.02-4.29)	4.23 (3.97-4.45)	4.23 (4.04-4.39)	4.18 (4.00-4.38)	4.16 (3.97-4.40)
LXN	1.29 (1.14-1.41)	1.26 (1.10-1.39)	1.25 (1.14-1.36)	1.29 (1.19-1.43)	1.31 (1.21-1.43)	1.31 (1.18-1.43)
MANF	6.59 (6.23-6.85)	6.59 (6.22-7.02)	6.45 (6.09-6.73)	6.34 (6.02-6.65)	6.38 (6.06-6.73)	6.44 (6.01-6.82)
MATN3	1.56 (1.38-1.89)	1.63 (1.41-1.88)	1.71 (1.47-1.97)	1.75 (1.49-1.92)	1.63 (1.40-1.90)	1.64 (1.42-1.85)
MDGA1	4.75 (4.01-5.21)	4.55 (4.12-5.05)	4.61 (3.91-5.16)	4.46 (4.08-5.09)	4.53 (4.02-5.13)	4.59 (3.93-4.92)
MSR1	4.30 (4.01-4.64)	4.28 (4.07-4.60)	4.40 (4.15-4.76)	4.26 (3.99-4.59)	4.23 (3.97-4.52)	4.35 (4.09-4.67)
N_CDase	2.41 (1.97-2.70)	2.35 (1.96-2.64)	2.45 (2.10-2.78)	2.36 (1.96-2.79)	2.29 (1.96-2.73)	2.39 (1.94-2.81)
N2DL_2	2.74 (2.56-2.95)	2.64 (2.41-2.91)	2.66 (2.47-2.86)	2.61 (2.45-2.83)	2.64 (2.43-2.85)	2.68 (2.50-2.87)
NAAA	2.62 (2.41-2.92)	2.52 (2.25-2.92)	2.51 (2.23-2.83)	2.50 (2.25-2.86)	2.43 (2.11-2.75)	2.47 (2.22-2.81)
NBL1	4.42 (4.34-4.50)	4.40 (4.32-4.47)	4.35 (4.26-4.44)	4.36 (4.28-4.49)	4.37 (4.31-4.46)	4.35 (4.27-4.45)
NCAN	6.78 (6.59-7.02)	6.77 (6.42-6.96)	6.87 (6.62-7.07)	6.85 (6.66-7.00)	6.87 (6.62-7.06)	6.87 (6.59-7.06)
NEP	1.73 (1.29-2.10)	1.96 (1.49-2.33)	1.78 (1.17-2.19)	1.81 (1.44-2.13)	1.83 (1.21-2.39)	1.88 (1.44-2.44)
NMNAT1	3.55 (2.86-4.16)	3.82 (3.17-4.40)	3.50 (3.15-3.97)	3.75 (3.33-4.38)	3.39 (2.86-3.95)	3.73 (3.16-4.27)
Nr_CAM	8.19 (8.04-8.32)	8.14 (8.09-8.25)	8.18 (8.04-8.33)	8.18 (8.04-8.24)	8.19 (8.05-8.29)	8.19 (8.04-8.27)

NRP2	2.78 (2.60-3.11)	2.88 (2.63-3.23)	2.61 (2.44-2.75)	2.63 (2.35-2.84)	2.67 (2.47-3.05)	2.70 (2.47-2.92)
NTRK2	4.77 (4.63-4.86)	4.73 (4.65-4.83)	4.70 (4.61-4.79)	4.69 (4.57-4.80)	4.70 (4.58-4.81)	4.72 (4.58-4.83)
NTRK3	6.60 (6.38-6.72)	6.56 (6.43-6.71)	6.57 (6.44-6.70)	6.53 (6.37-6.66)	6.52 (6.36-6.68)	6.52 (6.35-6.65)
PDGF_R_alpha	3.87 (3.70-4.04)	3.87 (3.65-4.06)	3.82 (3.66-3.99)	3.79 (3.62-3.93)	3.74 (3.57-3.99)	3.82 (3.61-3.99)
PLXNB3	3.79 (3.52-4.05)	3.79 (3.53-3.97)	3.60 (3.40-3.80)	3.52 (3.34-3.72)	3.62 (3.42-3.77)	3.59 (3.34-3.77)
PRTG	5.41 (5.21-5.55)	5.36 (5.19-5.49)	5.35 (5.21-5.48)	5.36 (5.14-5.48)	5.34 (5.20-5.53)	5.35 (5.20-5.53)
PVR	6.84 (6.67-7.00)	6.90 (6.71-7.11)	6.88 (6.64-7.10)	6.87 (6.70-7.11)	6.88 (6.66-7.10)	6.88 (6.69-7.06)
RGMA	7.59 (7.40-7.77)	7.58 (7.41-7.77)	7.44 (7.31-7.71)	7.47 (7.31-7.64)	7.55 (7.31-7.74)	7.52 (7.34-7.69)
RGMB	5.04 (4.84-5.25)	4.99 (4.88-5.29)	5.03 (4.86-5.27)	5.02 (4.91-5.24)	5.10 (4.86-5.35)	5.06 (4.93-5.29)
ROBO2	4.33 (4.06-4.57)	4.26 (4.02-4.41)	4.27 (4.05-4.48)	4.24 (3.99-4.40)	4.18 (4.00-4.36)	4.27 (4.03-4.47)
RSPO1	1.91 (1.70-2.12)	1.93 (1.71-2.07)	2.11 (1.93-2.26)	2.06 (1.88-2.29)	1.91 (1.74-2.09)	1.96 (1.76-2.16)
SCARA5	6.89 (6.74-7.03)	6.89 (6.78-7.03)	6.86 (6.73-6.96)	6.86 (6.67-6.97)	6.85 (6.64-6.98)	6.86 (6.69-7.01)
SCARB2	2.57 (2.41-2.73)	2.57 (2.43-2.70)	2.62 (2.42-2.80)	2.65 (2.45-2.79)	2.55 (2.36-2.76)	2.63 (2.48-2.81)
SCARF2	4.98 (4.84-5.16)	4.92 (4.72-5.15)	5.04 (4.81-5.30)	4.99 (4.73-5.15)	4.94 (4.73-5.12)	4.93 (4.76-5.17)
sFRP_3	2.67 (2.34-3.04)	2.42 (2.25-2.91)	3.43 (3.01-3.62)	3.23 (2.82-3.61)	3.08 (2.67-3.50)	3.06 (2.72-3.24)
Siglec_9	4.31 (4.03-4.56)	4.34 (4.13-4.54)	4.32 (4.05-4.59)	4.30 (4.08-4.54)	4.33 (4.07-4.50)	4.36 (4.07-4.65)
SIGLEC1	3.95 (3.73-4.20)	3.88 (3.61-4.15)	3.92 (3.50-4.29)	3.81 (3.56-4.12)	3.89 (3.59-4.18)	3.94 (3.67-4.28)
SKR3	7.07 (6.96-7.21)	7.08 (6.97-7.20)	7.08 (6.92-7.21)	7.04 (6.94-7.21)	6.99 (6.84-7.23)	7.11 (6.93-7.27)
SMOC2	8.22 (7.92-8.41)	8.18 (8.03-8.44)	8.15 (7.87-8.45)	8.18 (7.94-8.40)	8.14 (7.88-8.40)	8.19 (7.99-8.52)
SMPD1	3.91 (3.64-4.22)	3.88 (3.53-4.19)	3.94 (3.66-4.21)	3.90 (3.67-4.20)	3.85 (3.57-4.16)	3.89 (3.70-4.16)
SPOCK1	2.50 (2.23-2.64)	2.46 (2.29-2.62)	2.59 (2.41-2.79)	2.58 (2.40-2.77)	2.54 (2.39-2.72)	2.56 (2.36-2.69)
THY1	8.97 (8.80-9.11)	8.96 (8.80-9.12)	8.97 (8.82-9.13)	8.95 (8.83-9.12)	8.96 (8.79-9.13)	9.02 (8.85-9.13)
TMPRSS5	2.81 (2.65-3.05)	2.81 (2.44-2.93)	2.81 (2.65-3.07)	2.73 (2.48-2.88)	2.74 (2.55-3.02)	2.71 (2.46-2.93)
TN_R	3.53 (3.20-3.91)	3.45 (3.24-3.65)	3.34 (3.15-3.67)	3.34 (3.12-3.61)	3.37 (3.16-3.66)	3.46 (3.16-3.81)
TNFRSF12A	5.21 (4.91-5.47)	5.21 (5.03-5.41)	5.22 (4.86-5.40)	5.30 (5.00-5.47)	5.10 (4.85-5.40)	5.26 (5.00-5.61)
TNFRSF21	8.54 (8.34-8.66)	8.40 (8.28-8.51)	8.46 (8.31-8.65)	8.37 (8.20-8.54)	8.43 (8.29-8.61)	8.43 (8.31-8.62)
UNC5C	2.70 (2.44-2.92)	2.64 (2.49-2.81)	2.69 (2.52-2.97)	2.65 (2.43-2.82)	2.72 (2.43-2.86)	2.67 (2.44-2.92)
WFIKKN1	2.90 (2.63-3.13)	2.87 (2.68-3.08)	2.85 (2.66-3.12)	2.82 (2.60-3.04)	2.92 (2.68-3.12)	2.94 (2.75-3.12)
VWC2	3.93 (3.66-4.32)	3.86 (3.59-4.14)	3.91 (3.65-4.22)	3.94 (3.68-4.22)	3.84 (3.54-4.14)	3.85 (3.55-4.13)
