# Supplementary material for: Exposure to Mild Steel Welding and Changes in Serum Proteins With Putative Neurological Function—A Longitudinal Study
Source: Front Public Health. 2020 Aug 28;8:422. doi: 10.3389/fpubh.2020.00422 (PMC7485227; doi:10.3389/fpubh.2020.00422)
Supplement: Supplementary Table 3 — Complete output of the differential protein expression analysis between welders and controls in the longitudinal study group (linear mixed models) and corresponding data for the cross-sectional group (linear models). [file Table_3.pdf]

Supplementary Table 3. Complete output of the differential protein expression analysis between welders and controls in the longitudinal study group (linear mixed models) and corresponding data for the cross-sectional group (linear models).

Protein	Linear Mixed Models (n=246)			Linear models (cross-sectional group) (n=191)		
	R_m^2 (%)[a]	Beta (SE)[b]	p[c]	R^2 (%)[d]	Beta (SE)[e]	p[f]
TNFRSF21	8	-0.112 (0.039)	0.004	4	-0.008 (0.037)	0.838
TMPRSS5	7	-0.171 (0.059)	0.004	-1	-0.033 (0.056)	0.554
NEP	10	0.257 (0.116)	0.027	10	-0.022 (0.102)	0.833
GDF_8	6	0.185 (0.087)	0.033	5	0.066 (0.072)	0.365
NMNAT1	2	0.256 (0.126)	0.043	7	0.28 (0.129)	0.032
gal_8	12	-0.122 (0.063)	0.052	0	-0.061 (0.058)	0.296
SCARF2	10	-0.086 (0.046)	0.064	13	0.012 (0.048)	0.797
GDNFR_alpha_3	2	-0.079 (0.044)	0.074	2	-0.039 (0.039)	0.309
IL12	4	-0.193 (0.109)	0.076	6	-0.006 (0.089)	0.945
JAM_B	4	-0.08 (0.047)	0.089	2	0.042 (0.046)	0.359
NCAN	10	-0.095 (0.056)	0.091	8	0.017 (0.055)	0.759
Alpha_2_MRAP	7	-0.127 (0.075)	0.093	6	-0.112 (0.111)	0.312
CTSC	4	0.102 (0.067)	0.127	6	-0.111 (0.06)	0.069
ROBO2	5	-0.073 (0.049)	0.131	5	0.026 (0.05)	0.598
CDH3	8	-0.082 (0.057)	0.153	2	0.028 (0.053)	0.604
UNC5C	3	-0.068 (0.051)	0.183	3	-0.018 (0.051)	0.722
CLM_6	2	-0.053 (0.042)	0.206	7	-0.023 (0.037)	0.530
EPHB6	13	-0.057 (0.046)	0.210	0	-0.018 (0.045)	0.692
CADM3	3	-0.094 (0.076)	0.215	-2	-0.003 (0.067)	0.961
CD200	4	-0.06 (0.052)	0.250	-1	-0.035 (0.045)	0.438
NRP2	2	0.065 (0.059)	0.264	1	-0.093 (0.077)	0.230
CRTAM	7	0.097 (0.087)	0.267	2	0.074 (0.08)	0.356
PLXNB3	6	-0.055 (0.049)	0.267	0	-0.051 (0.049)	0.295
Nr_CAM	12	-0.027 (0.025)	0.282	9	-0.041 (0.027)	0.122
GCP5	9	-0.114 (0.109)	0.292	4	-0.22 (0.095)	0.021
sFRP_3	13	-0.093 (0.089)	0.296	-1	-0.054 (0.084)	0.521
CLEC1B	5	0.072 (0.07)	0.302	3	-0.07 (0.073)	0.339
PVR	4	0.055 (0.053)	0.303	1	-0.025 (0.047)	0.589
SMOC2	5	0.071 (0.069)	0.303	6	0.039 (0.057)	0.491
CLM_1	1	-0.091 (0.089)	0.306	4	-0.023 (0.087)	0.794
FLRT2	5	-0.044 (0.044)	0.312	7	0.012 (0.04)	0.767
EZR	4	0.036 (0.036)	0.314	17	0.095 (0.036)	0.009
N2DL_2	6	-0.054 (0.054)	0.316	2	-0.022 (0.06)	0.713
PDGF_R_alpha	1	-0.041 (0.042)	0.326	1	0.025 (0.043)	0.556
SIGLEC1	7	-0.07 (0.079)	0.377	12	-0.02 (0.076)	0.798
ADAM22	8	-0.052 (0.06)	0.390	3	-0.007 (0.056)	0.901
LAYN	2	-0.047 (0.056)	0.402	5	0.019 (0.048)	0.693
DRAXIN	4	-0.055 (0.069)	0.421	-1	-0.007 (0.066)	0.919
GZMA	2	-0.079 (0.103)	0.443	3	0 (0.054)	0.994
CNTN5	1	0.049 (0.065)	0.452	-1	-0.033 (0.065)	0.612
SCARA5	6	0.028 (0.037)	0.453	11	-0.01 (0.036)	0.780
LAIR_2	2	-0.13 (0.192)	0.498	-1	-0.017 (0.168)	0.918
NTRK3	1	-0.026 (0.039)	0.504	2	-0.009 (0.036)	0.800
PRTG	5	-0.03 (0.046)	0.505	1	-0.025 (0.043)	0.569
CD38	2	-0.039 (0.059)	0.515	3	0.069 (0.057)	0.225
CTSS	8	0.024 (0.037)	0.522	12	-0.107 (0.034)	0.002
FcRL2	7	0.048 (0.075)	0.524	2	-0.003 (0.068)	0.967
WFIKKN1	11	-0.033 (0.052)	0.526	8	-0.011 (0.055)	0.845
NAAA	1	-0.05 (0.08)	0.531	0	0.071 (0.076)	0.350

SCARB2	10	0.024 (0.038)	0.531	19	0.055 (0.04)	0.167
KYNU	16	0.058 (0.095)	0.543	19	-0.053 (0.088)	0.548
LAT	1	-0.043 (0.072)	0.548	2	-0.104 (0.071)	0.143
Beta_NGF	9	-0.021 (0.035)	0.548	2	-0.076 (0.048)	0.111
VWC2	6	-0.042 (0.07)	0.550	8	-0.027 (0.066)	0.682
DDR1	2	0.021 (0.035)	0.552	2	-0.022 (0.033)	0.518
TNFRSF12A	0	0.034 (0.059)	0.563	5	0.145 (0.064)	0.024
CD200R1	5	-0.033 (0.058)	0.572	3	0.029 (0.057)	0.619
EDA2R	30	-0.038 (0.069)	0.578	23	0.004 (0.07)	0.960
BCAN	9	-0.029 (0.055)	0.602	5	0.021 (0.055)	0.709
MANF	3	0.043 (0.085)	0.611	3	-0.016 (0.085)	0.850
CPA2	2	-0.044 (0.086)	0.613	-1	-0.057 (0.087)	0.517
IL_5R_alpha	10	-0.048 (0.097)	0.623	9	-0.13 (0.093)	0.162
RSPO1	15	-0.026 (0.053)	0.628	9	0.023 (0.045)	0.618
GM_CSF_R_alpha	2	-0.055 (0.126)	0.661	0	0.088 (0.115)	0.444
NTRK2	7	-0.011 (0.026)	0.663	12	-0.017 (0.026)	0.511
GFR_alpha_1	6	-0.019 (0.043)	0.664	7	-0.009 (0.04)	0.818
LXN	4	0.011 (0.026)	0.671	3	-0.021 (0.028)	0.471
TN_R	13	-0.026 (0.063)	0.677	-1	0.043 (0.069)	0.529
Siglec_9	1	0.021 (0.057)	0.710	1	0.062 (0.05)	0.214
ADAM23	14	-0.03 (0.087)	0.730	1	-0.093 (0.086)	0.278
CLEC10A	0	0.022 (0.074)	0.769	1	0.087 (0.068)	0.203
Dkk_4	2	0.016 (0.055)	0.776	1	-0.052 (0.06)	0.389
MDGA1	3	-0.033 (0.122)	0.788	-1	-0.062 (0.104)	0.554
RGMA	17	0.012 (0.047)	0.792	4	-0.001 (0.043)	0.982
MSR1	23	-0.017 (0.067)	0.800	20	0.046 (0.062)	0.464
SMPD1	5	-0.015 (0.069)	0.830	4	0.073 (0.062)	0.242
G_CSF	2	0.014 (0.069)	0.845	3	0.04 (0.074)	0.588
CDH6	5	0.009 (0.046)	0.851	1	-0.004 (0.04)	0.914
MATN3	2	-0.01 (0.055)	0.863	-1	-0.042 (0.053)	0.425
N_CDase	10	-0.016 (0.096)	0.865	12	-0.008 (0.077)	0.920
SPOCK1	7	0.007 (0.042)	0.867	2	-0.027 (0.038)	0.486
THY1	10	0.006 (0.035)	0.869	13	0 (0.035)	0.994
RGMB	5	-0.006 (0.047)	0.895	1	0.001 (0.047)	0.984
EFNA4	2	-0.004 (0.033)	0.898	15	0.01 (0.036)	0.781
NBL1	5	-0.002 (0.027)	0.927	1	-0.039 (0.025)	0.119
CPM	11	-0.002 (0.032)	0.944	18	0.002 (0.035)	0.955
SKR3	2	0.002 (0.036)	0.945	11	0.055 (0.036)	0.126

SE, standard error; [a]Variance explained by fixed factors (group, age, body-mass index); [b]regression coefficient from linear mixed models interpreted as standard deviation difference in protein levels compared to controls, adjusted for age, body-mass index variables as fixed factors, and participant as random factors; [c]P-value from test of contribution of group inclusion (welders and controls) to protein variance using an analysis of variance approach with Satterthwaite approximation for degrees of freedom (Bonferroni-adjusted threshold for the p-value: $0.05/87 = 5.7*10^{-4}$); [d]variance in protein levels explained by the linear model; [e]regression coefficient from multivariable-adjusted linear models interpreted as standard deviation difference in protein levels compared to controls adjusted for age, body-mass index; [f]p-value from the linear model to test the difference between welders and control;
