# Supplementary material for: Exposure to Mild Steel Welding and Changes in Serum Proteins With Putative Neurological Function—A Longitudinal Study
Source: Front Public Health. 2020 Aug 28;8:422. doi: 10.3389/fpubh.2020.00422 (PMC7485227; doi:10.3389/fpubh.2020.00422)
Supplement: Supplementary Table 4 — Gene ontologies (biological process category) related to proteins that were differentially expressed in both the longitudinal and the cross-sectional analyses (according to uniprot.org). [file Table_4.pdf]

Supplementary Table 4. Gene ontologies (biological process category) related to proteins that were differentially expressed in both the longitudinal and the cross-sectional analyses (according to uniprot.org).

**Protein**	**GO term (Biological process)**
NMNAT1	ATP generation from poly-ADP-D-ribose<br>NAD biosynthetic process<br>NAD metabolic process<br>negative regulation of apoptotic DNA fragmentation<br>negative regulation of neuron apoptotic process<br>nucleotide biosynthetic process<br>positive regulation of MAPK cascade<br>response to wounding
GCSF	cellular response to cytokine stimulus<br>cellular response to lipopolysaccharide<br>cytokine-mediated signaling pathway<br>granulocyte differentiation<br>immune response<br>multicellular organism development<br>negative regulation of neuron death<br>positive regulation of actin cytoskeleton reorganization<br>positive regulation of actin filament polymerization<br>positive regulation of cell population proliferation<br>positive regulation of DNA-binding transcription factor activity<br>positive regulation of myeloid cell differentiation<br>positive regulation of peptidyl-serine phosphorylation<br>positive regulation of peptidyl-tyrosine phosphorylation<br>positive regulation of phosphatidylinositol 3-kinase signaling<br>positive regulation of protein binding<br>positive regulation of protein kinase B signaling<br>positive regulation of transcription by RNA polymerase II<br>response to ethanol
EFNA4	axon guidance<br>bone remodeling<br>cell-cell signaling<br>ephrin receptor signaling pathway<br>osteoclast differentiation
CTSS	adaptive immune response<br>antigen processing and presentation<br>antigen processing and presentation of exogenous peptide antigen via MHC class II<br>antigen processing and presentation of peptide antigen<br>basement membrane disassembly<br>cellular response to thyroid hormone stimulus<br>collagen catabolic process<br>extracellular matrix disassembly<br>immune response<br>neutrophil degranulation<br>positive regulation of cation channel activity<br>protein processing<br>proteolysis<br>proteolysis involved in cellular protein catabolic process<br>response to acidic pH<br>toll-like receptor signaling pathway
CLM6	cellular defense response<br>immune system process Source<br>regulation of immune response Source
VWC2	negative regulation of BMP signaling pathway<br>positive regulation of cell-substrate adhesion<br>positive regulation of neuron differentiation
