# Supplementary material for: Exposure to Mild Steel Welding and Changes in Serum Proteins With Putative Neurological Function—A Longitudinal Study
Source: Front Public Health. 2020 Aug 28;8:422. doi: 10.3389/fpubh.2020.00422 (PMC7485227; doi:10.3389/fpubh.2020.00422)
Supplement: Supplementary Table 5 — Associations of the significantly differentially expressed proteins in welders and controls with exposure to welding expressed as respirable dust (adjusted for personal respiratory protection equipment), years welding and cumulative exposure in welders only (linear mixed models). [file Table_5.pdf]

Supplementary Table 5. Associations of the significantly differentially expressed proteins in welders and controls with exposure to welding expressed as respirable dust (adjusted for personal respiratory protection equipment), years welding and cumulative exposure in welders only (linear mixed models).

Protein	Respirable dust adjusted (n=84)			Years welding (n=112)			Cumulative exposure (n=84)		
	R_m^2 (%)[a]	Beta (SE)[b]	p[c]	R_m^2 (%)[a]	Beta (SE)[b]	p[c]	R_m^2 (%)[a]	Beta (SE)[b]	p[c]
TNFRSF21	4	-0.011 (0.022)	0.613	2	-0.001 (0.004)	0.820	4	0.001 (0.003)	0.690
TMPRSS5	9	-0.030 (0.025)	0.228	8	0.004 (0.005)	0.389	11	-0.005 (0.003)	0.148
NEP	13	0.128 (0.070)	0.067	11	-0.015 (0.012)	0.210	11	-0.003 (0.008)	0.688
GDF8	3	0.008 (0.054)	0.888	5	0.000 (0.008)	0.984	3	0.002 (0.006)	0.675
NMNAT1	11	0.008 (0.093)	0.933	2	-0.005 (0.013)	0.683	11	-0.002 (0.008)	0.807

SE, standard error; [a]Variance explained by fixed factors (respirable dust/years welding/cumulative exposure, age, body-mass index); [b]regression coefficient from linear mixed models interpreted as standard deviation difference in protein levels per respirable dust unit increase/numbers of years welding/cumulative exposure unit increase adjusted for age, body-mass index variables as fixed factors, and participant as random factors; [c]p-value from test of contribution of respirable dust/years welding/cumulative exposure to protein variance using an analysis of variance approach with Satterthwaite approximation for degrees of freedom.
