# Supplementary material for: Exposure to Mild Steel Welding and Changes in Serum Proteins With Putative Neurological Function—A Longitudinal Study
Source: Front Public Health. 2020 Aug 28;8:422. doi: 10.3389/fpubh.2020.00422 (PMC7485227; doi:10.3389/fpubh.2020.00422)
Supplement: Supplementary Table 6 — Proteins associated with age in the longitudinal study group (linear mixed models) and corresponding data for the cross-sectional group (linear models). [file Table_6.pdf]

**Supplementary Table 6. Proteins associated with age in the longitudinal study group (linear mixed models) and corresponding data for the cross-sectional group (linear models).**

| | Linear mixed models (n=246) | | | Linear models (n=191) | | |
|---|---|---|---|---|---|---|
| **Protein** | $R_m^2$ (%)[a] | Beta (SE)[b] | p[c] | $R^2$ (%)[d] | Beta (SE)[e] | p[f] |
| EDA2R | 29 | 0.022 (0.003) | <0.001[#] | 23 | 0.023 (0.003) | <0.001# |
| RGMA | 17 | -0.011 (0.002) | <0.001# | 5 | -0.007 (0.002) | 0.001 |
| ADAM23 | 14 | -0.02 (0.004) | <0.001# | 0 | -0.006 (0.004) | 0.115 |
| RSPO1 | 14 | 0.012 (0.002) | <0.001# | 9 | 0.008 (0.002) | <0.001# |
| EPHB6 | 12 | -0.009 (0.002) | <0.001# | 1 | -0.003 (0.002) | 0.197 |
| GCP5 | 8 | -0.017 (0.004) | <0.001# | 2 | -0.011 (0.005) | 0.015 |
| WFIKKN1 | 11 | -0.01 (0.002) | <0.001# | 9 | -0.009 (0.003) | 0.001 |
| MSR1 | 23 | 0.013 (0.003) | <0.001# | 20 | 0.013 (0.003) | <0.001# |
| sFRP_3 | 13 | 0.018 (0.004) | <0.001# | 0 | 0.001 (0.004) | 0.822 |
| Beta_NGF | 9 | -0.007 (0.002) | <0.001# | 1 | 0.001 (0.002) | 0.625 |
| TN_R | 13 | -0.011 (0.003) | <0.001# | -1 | -0.003 (0.003) | 0.375 |
| BCAN | 9 | -0.01 (0.002) | <0.001# | 6 | -0.007 (0.003) | 0.010 |
| SCARF2 | 8 | 0.008 (0.002) | <0.001# | 14 | 0.012 (0.002) | <0.001# |
| gal_8 | 10 | -0.01 (0.003) | <0.001# | 0 | -0.003 (0.003) | 0.244 |
| CTSS | 8 | -0.006 (0.002) | <0.001# | 7 | -0.006 (0.002) | 0.001 |
| Nr_CAM | 11 | -0.004 (0.001) | <0.001# | 8 | -0.003 (0.001) | 0.009 |
| IL_5R_alpha | 10 | 0.012 (0.004) | <0.001# | 9 | 0.017 (0.004) | <0.001# |
| CDH3 | 6 | -0.008 (0.002) | <0.001# | 3 | -0.006 (0.003) | 0.011 |
| CRTAM | 6 | -0.012 (0.003) | 0.001 | 2 | -0.005 (0.004) | 0.146 |
| CDH6 | 5 | -0.006 (0.002) | 0.001 | 2 | 0 (0.002) | 0.858 |
| PLXNB3 | 5 | -0.007 (0.002) | 0.002 | 0 | -0.002 (0.002) | 0.481 |
| RGMB | 5 | -0.006 (0.002) | 0.002 | 2 | -0.005 (0.002) | 0.028 |
| NBL1 | 5 | -0.003 (0.001) | 0.003 | 0 | -0.002 (0.001) | 0.180 |
| ADAM22 | 7 | -0.007 (0.002) | 0.006 | 4 | -0.001 (0.003) | 0.795 |
| FcRL2 | 7 | -0.007 (0.003) | 0.006 | 2 | 0.007 (0.003) | 0.038 |
| CLEC1B | 5 | -0.008 (0.003) | 0.008 | 3 | -0.003 (0.003) | 0.396 |
| CD200R1 | 5 | -0.006 (0.002) | 0.009 | 4 | -0.002 (0.003) | 0.358 |
| CD200 | 3 | -0.005 (0.002) | 0.010 | -1 | 0 (0.002) | 0.864 |
| NCAN | 8 | -0.006 (0.002) | 0.011 | 9 | -0.006 (0.003) | 0.019 |
| SMOC2 | 5 | -0.007 (0.003) | 0.017 | 6 | -0.006 (0.003) | 0.042 |
| N2DL_2 | 5 | -0.005 (0.002) | 0.019 | 2 | 0.003 (0.003) | 0.225 |
| PRTG | 4 | -0.004 (0.002) | 0.020 | 1 | -0.003 (0.002) | 0.089 |
| SMPD1 | 5 | 0.006 (0.003) | 0.024 | 3 | 0.007 (0.003) | 0.024 |
| SCARB2 | 10 | 0.004 (0.002) | 0.025 | 19 | 0.009 (0.002) | <0.001 |
| ROBO2 | 4 | -0.005 (0.002) | 0.026 | 5 | -0.006 (0.002) | 0.014 |
| FLRT2 | 5 | -0.004 (0.002) | 0.027 | 7 | -0.001 (0.002) | 0.726 |
| TNFRSF21 | 3 | -0.004 (0.002) | 0.029 | 4 | 0.002 (0.002) | 0.348 |
| CTSC | 3 | -0.006 (0.003) | 0.039 | 4 | 0.004 (0.003) | 0.220 |

SE, standard error; [a]Variance explained by fixed factors (age, body-mass index); [b]regression coefficient from linear mixed models interpreted as standard deviation difference in protein levels per age (years) adjusted body-mass index variables as fixed factors, and participant as random factors; [c]p-value from test of contribution of age years to protein variance using an analysis of variance approach with Satterthwaite approximation for degrees of freedom (Bonferroni-adjusted threshold for the p-value: 0.05/87 = $5.7*10^{-4}$); [d]variance in protein levels explained by the linear model; [e]regression coefficient from linear mixed models interpreted as standard deviation difference in protein levels per age (years) adjusted for body-mass index variables; [f]p-value from the linear model to test the association with age; [#]significant after adjustment for multiple testing (Bonferroni); only proteins significantly associated with age in the linear mixed models are included in this table.
