# Supplementary material for: Exposure to Mild Steel Welding and Changes in Serum Proteins With Putative Neurological Function—A Longitudinal Study
Source: Front Public Health. 2020 Aug 28;8:422. doi: 10.3389/fpubh.2020.00422 (PMC7485227; doi:10.3389/fpubh.2020.00422)
Supplement: Supplementary Table 7 — Proteins associated with BMI in the longitudinal study group (linear mixed models) and corresponding data for the cross-sectional group (linear models). [file Table_7.pdf]

Supplementary Table 7. Proteins associated with BMI in the longitudinal study group (linear mixed models) and corresponding data for the cross-sectional group (linear models).

Protein	Adjusted AGE					
	Linear mixed models (n=246)			Linear models (n=191)		
	R_m^2 (%)[a]	Beta (SE)[b]	p[c]	R^2 (%)[d]	Beta (SE)[e]	p[f]
KYNU	16	0.062 (0.011)	<0.001#	20	0.072 (0.011)	<0.001#
MSR1	23	0.034 (0.007)	<0.001#	20	0.034 (0.007)	<0.001#
CPM	11	0.017 (0.004)	<0.001#	18	0.022 (0.004)	<0.001#
THY1	10	0.017 (0.004)	<0.001#	13	0.023 (0.004)	<0.001#
N_CDase	10	0.04 (0.009)	<0.001#	12	0.045 (0.009)	<0.001#
NEP	8	0.049 (0.012)	<0.001#	10	0.059 (0.012)	<0.001#
TN_R	13	0.027 (0.007)	<0.001#	-1	0.002 (0.008)	0.786
Nr_CAM	11	0.01 (0.003)	<0.001#	8	0.013 (0.003)	<0.001#
FcRL2	7	0.024 (0.007)	<0.001#	2	0.007 (0.008)	0.358
SCARB2	10	0.015 (0.004)	<0.001#	19	0.017 (0.005)	<0.001#
NTRK2	7	0.011 (0.003)	<0.001#	12	0.016 (0.003)	<0.001#
SCARA5	6	0.013 (0.004)	0.001	12	0.02 (0.004)	<0.001#
ADAM22	7	0.021 (0.006)	0.001	4	0.02 (0.007)	0.003
SIGLEC1	7	0.026 (0.008)	0.001	13	0.038 (0.009)	<0.001#
gal_8	10	0.02 (0.006)	0.002	0	0.008 (0.007)	0.226
GFR_alpha_1	5	0.015 (0.005)	0.002	8	0.02 (0.005)	<0.001#
MDGA1	3	0.027 (0.009)	0.003	0	0.012 (0.012)	0.320
VWC2	6	0.021 (0.007)	0.003	9	0.022 (0.008)	0.007
SPOCK1	7	0.014 (0.005)	0.004	2	0.009 (0.005)	0.040
Alpha_2_MRAP	5	0.02 (0.007)	0.005	6	0.039 (0.013)	0.003
N2DL_2	5	0.015 (0.005)	0.005	2	0.014 (0.007)	0.053
PVR	4	0.015 (0.005)	0.005	1	0.011 (0.006)	0.048
EZR	4	0.011 (0.004)	0.006	14	0.025 (0.004)	<0.001#
LAIR_2	1	0.03 (0.011)	0.006	0	-0.003 (0.02)	0.887
IL_5R_alpha	10	0.024 (0.009)	0.007	9	0.015 (0.011)	0.192
EDA2R	29	0.019 (0.007)	0.007	23	0.017 (0.008)	0.048
NCAN	8	-0.017 (0.006)	0.008	9	-0.02 (0.007)	0.002
DRAXIN	4	0.019 (0.008)	0.013	-1	0.003 (0.008)	0.714
GDF_8	4	0.022 (0.009)	0.014	5	0.024 (0.009)	0.006
FLRT2	5	0.011 (0.005)	0.016	7	0.02 (0.005)	<0.001#
SMOC2	5	0.017 (0.007)	0.017	6	0.025 (0.007)	<0.001#
CD200R1	5	0.013 (0.006)	0.022	4	0.021 (0.007)	0.003
PRTG	4	0.01 (0.005)	0.025	1	0.008 (0.005)	0.115
JAM_B	3	0.011 (0.005)	0.026	2	0.011 (0.006)	0.039
EFNA4	3	0.008 (0.004)	0.027	15	0.022 (0.004)	<0.001#
MANF	3	0.019 (0.009)	0.034	3	0.03 (0.01)	0.004
sFRP_3	13	0.022 (0.01)	0.038	0	0.011 (0.01)	0.271
GM_CSF_R_alpha	2	0.021 (0.01)	0.039	0	0.014 (0.014)	0.299
WFIKKN1	11	0.011 (0.005)	0.040	9	0.024 (0.007)	<0.001#
CRTAM	6	0.018 (0.009)	0.045	2	0.02 (0.01)	0.037
SMPD1	5	0.014 (0.007)	0.049	3	0.01 (0.007)	0.195

SE, standard error; [a]Variance explained by fixed factors (age, body-mass index); [b]regression coefficient from linear mixed models interpreted as standard deviation difference in protein levels per BMI adjusted for age variables as fixed factors, and participant as random factors; [c]p-value from test of contribution of BMI to protein variance using an analysis of variance approach with Satterthwaite approximation for degrees of freedom (Bonferroni-adjusted threshold for the p-value: 0.05/87 = $5.7*10^{-4}$); [d]variance in protein levels explained by the linear model; [e]regression coefficient from linear mixed models interpreted as standard deviation difference in protein levels per BMI adjusted for age; [f]p-value from the linear model to test the association with BMI; [#]significant after adjustment for multiple testing (Bonferroni); only proteins significantly associated with BMI in the linear mixed models are included in this table.
